# Instant Energy Barrier Modulation in Bistable Robotic Grippers for Compliant Triggering and Powerful Grasping

**DOI:** 10.34133/research.0737

**Published:** 2025-06-19

**Authors:** Jie Zhang, Hao Yang, Chenyu He, Hanfei Ma, Yuwen Zhao, Zongyu Zhang, Shengming Li, Wei Wang, Jinzhao Yang, Jianing Wu, Haijun Peng

**Affiliations:** ^1^ School of Mechanics and Aerospace Engineering, Dalian University of Technology, Dalian, China.; ^2^ State Key Laboratory of Structural Analysis, Optimization and CAE Software for Industrial Equipment, Dalian University of Technology, Dalian, China.; ^3^ School of Aeronautics and Astronautics, Sun Yat-Sen University, Shenzhen, China.; ^4^ Dalian Maritime University, College of Artificial Intelligence, Dalian, China.; ^5^ School of Innovation and Entrepreneurship, Dalian University of Technology, Dalian, China.; ^6^ School of Engineering, Westlake University, Hangzhou, China.; ^7^ Research Center for Industries of the Future, Westlake University, Hangzhou, China.; ^8^ School of Advanced Manufacturing, Sun Yat-Sen University, Shenzhen, China.

## Abstract

Bistable structures, which leverage mechanical instability, have emerged as a promising paradigm in the development of robotic grippers, providing advantages including rapid response and low energy consumption. A critical limitation of existing bistable grippers, however, lies in their invariable energy barriers, which hinder the balance between compliant triggering and powerful grasping. In this study, we propose a bistable robotic gripper capable of in situ energy barrier modulation, inspired by the adaptive seed dispersal behavior of *Impatiens* pods. This robotic gripper features an elastic curved beam-based architecture integrated with a motor-driven mechanism, enabling dynamic regulation of its energy landscape. This approach allows the energy barrier to be tuned over an order of magnitude during manipulation. In the low-barrier state, the robotic gripper initiates object interaction with a triggering force as low as 0.66 N, allowing for delicate manipulation. Upon state transition, instant energy barrier modulation (~300 ms) enhances grasping stability, achieving failure forces up to 12.08 N. This adaptive modulation strategy enables our robotic gripper to implement rapid, compliant, and powerful interaction. When incorporated into an unmanned aerial vehicle, the robotic gripper showcases reliable perching across diverse scenarios, highlighting the potential of energy barrier modulation to advance the adaptability and functionality of robotic systems.

## Introduction

Grasping behavior is a fundamental capability in biological organisms, essential for interacting with the environments in daily life [1]. Over the past decades, substantial efforts have been dedicated to developing robotic grippers to diminish reliance on labor-intensive tasks traditionally implemented by humans [2–5]. Despite these achievements, minimizing system complexity and enhancing operational controllability are persistent challenges [6]. To address this defect, researchers have explored robotic grippers that incorporate flexible materials and compliant structures, such as silicone rubber [7–9], origami [10–12], and kirigami [13–15]. These designs typically leverage passive adaptability, providing substantial potential for advancing robotic technologies [16]. However, these soft robotic grippers suffer from slow response speeds and a continuous need for energy input to maintain a grasp, thereby reducing their efficiency in practical applications [17].

In tackling these challenges, bistable structures have been introduced into robotic grippers as a viable solution. Recent advances have explored various bistable structures, ranging from one-dimensional (1D) beams to 3D mechanisms, providing substantial diversity in size and shape [18]. Bistable structures arise from mechanical instabilities and showcase 2 distinct stable equilibrium states [19–21], corresponding to open and closed configurations in robotic grippers [22–25]. Transitions between the stable states occur via snap-through action, enabling rapid displacement and morphing within a few hundred milliseconds [26,27]. Additionally, bistability demonstrate an energy-efficient mechanism, allowing robotic grippers to maintain a grasp passively without continuous energy input [28]. One notable application of these robotic grippers is their integration with unmanned aerial vehicles (UAVs), enabling perching on tree branches for extended durations, enhancing operational efficiency [29]. Beyond robotic grippers, the ability of bistable structures to generate both high-output force and power amplification makes them valuable in diverse robotic systems [30,31], such as swimmers [32–34], crawlers [35–37], and jumpers [38–40].

**Fig. 1.**
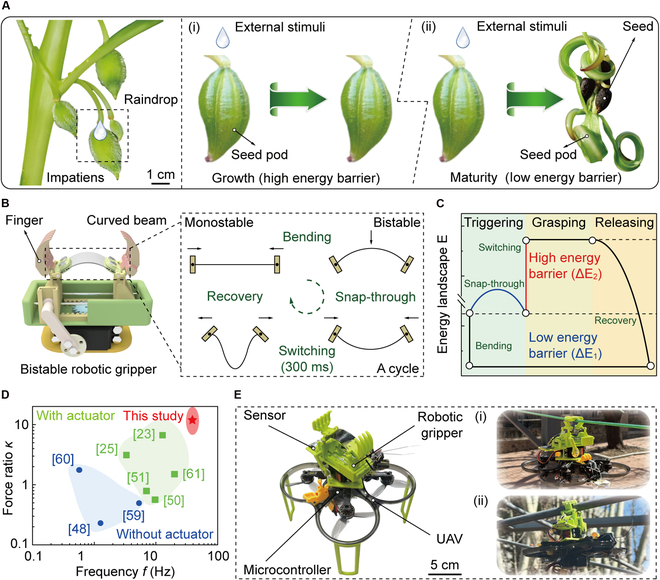
Bio-inspired bistable robotic grippers with tunable energy barriers. (A) Mature seed pods from plants in the genus *Impatiens*, demonstrating high sensitivity through the reduction of energy barriers. The seed pods with low energy barriers explosively release seeds in response to external stimuli, facilitating seed propagation. (B) Bistable robotic grippers facilitated by curved beams. By tuning the curvature of the beam, the energy barrier of the system can be precisely tuned, allowing the gripper to transition between compliant and powerful grasping behaviors. (C) Energy landscape of the bistable robotic gripper. (D) Performance comparison between our paradigm and existing bistable robotic grippers [23,25,48,50,51,59–61]. Here, *f* denotes the frequency of stable state transition, and *κ* represents the ratio between failure and triggering forces during grasping process. (E) Application demonstrations of bistable robotic grippers. Robotic gripper is integrated into UAVs for performing tasks such as grasping and perching, showing its potential for diverse functional uses.

Despite these advancements, conventional bistable structures always showcase specific deformation characteristics determined by their mechanical configurations as their energy barriers are constant. The invariable restricts the adaptability and performance optimizations after the initial setup [41]. In contrast, natural organisms show the ability to dynamically modulate their energy barriers, which enhances their adaptability to varying environmental stimuli. An striking example of this is observed in the seed pods of plants belonging to the genus *Impatiens*, which exhibit remarkable energy barrier modulation throughout their maturation process [42]. Initially, the seed pods maintain a relatively high energy barrier to prevent premature seed dispersal during growth. Upon maturity, the energy barrier is reduced, rendering the seed pods highly sensitive to external stimuli. In this state, minimal forces, such as raindrop impact, can trigger rapid explosive dehiscence, which facilitates efficient seed dispersal (Fig. 1A). This natural mechanism exemplifies the functional advantages of tunable energy barriers, which inspire the development of adaptive engineered systems [40].

In robotic grasping applications, particularly when manipulating fragile or delicate object, a low energy barrier is crucial for compliant triggering, as it minimizes the risk of damage during state transition. Once a secure grasp is established, increasing the energy barrier enhances stability, effectively preventing unintended release. This capability is essential for the trade-off between flexibility and stability throughout the grasping process. Hence, modulating energy barriers in bistable structures is vital for achieving rapid, compliant, and powerful grasping behaviors [43]. At present, one approach to achieving tunable energy barriers involves the use of thermally responsive materials, for example, shape memory alloys, which allows the modulation of energy barrier via temperature variations [44,45]. However, this approach introduces delays in response time due to thermal cycling, which compromises the dynamic performance of robotic grippers [46]. To address the limitation, alternative strategies have been explored, such as embedding bistable structures with magnetic microparticles, allowing energy barriers to be dynamically tuned via an external magnetic field [43]. This approach provides faster response times and greater precision in regulating bistable behavior. However, the reliance on an external magnetic field imposes environmental constraints, which may limit the applicability in certain real-world scenarios.

In this study, we propose an elastic beam-enabled bistable robotic gripper that achieves instantaneously tunable energy barriers in situ through dynamic curvature regulation of the beam, as shown in Fig. 1B. Initially, the monostable beam is bent upward, showing bistable behavior with an initial energy barrier of Δ*E*_1_. Upon the application of external forces, a snap-through phenomenon occurs, reconfiguring the beam into a down-curved stable state to implement grasping behaviors within 25 ms (switching frequency *f* = 40 Hz). Following the state transition, the energy barrier rises to Δ*E*_2_ by actuating the motor to program the curvature rapidly (~300 ms), enhancing the strength and stability during grasping. Then, mechanical models are derived to characterize this process, with the corresponding energy landscape demonstrated in Fig. 1C. Benefited from the tunability of the energy barrier, experimental results reveal that the ratio *κ* between triggering and failure forces goes up to 18.30, outperforming current bistable robotic grippers and demonstrating a capability for compliant triggering and powerful grasping (Fig. 1D). Moreover, we integrate our robotic gripper into UAVs for both grasping and perching tasks, enhancing its potential in diverse scenarios, as shown in Fig. 1E.

## Results

### General design and operational principle

To successfully perform a grasping task, bistable robotic grippers are required to exhibit a combination of diverse capabilities throughout the manipulation, including flexibility for triggering, rapidity for switching, and stability for grasping. To satisfy the multifaceted requirements, we develop a robotic gripper (*m* = 132.21 g) that seamlessly integrates these capabilities by leveraging a bistable elastic beam with an in situ tunable energy barrier (Fig. 1B and Fig. S1). In this design, the 2 ends of the beam are mounted on clamps, which are connected to structural supports via rotating shafts. Here, the fingers customized to meet specific application requirements can be inserted into these clamps. To achieve real-time modulation of the energy barrier, a motor-driven slider-crank mechanism is used. Moreover, the design features 2 synchronized racks positioned at the base of the supports. These racks move in opposite directions through a gear-driven mechanism, ensuring coordinated movement of both supports using a single motor.

**Fig. 2.**
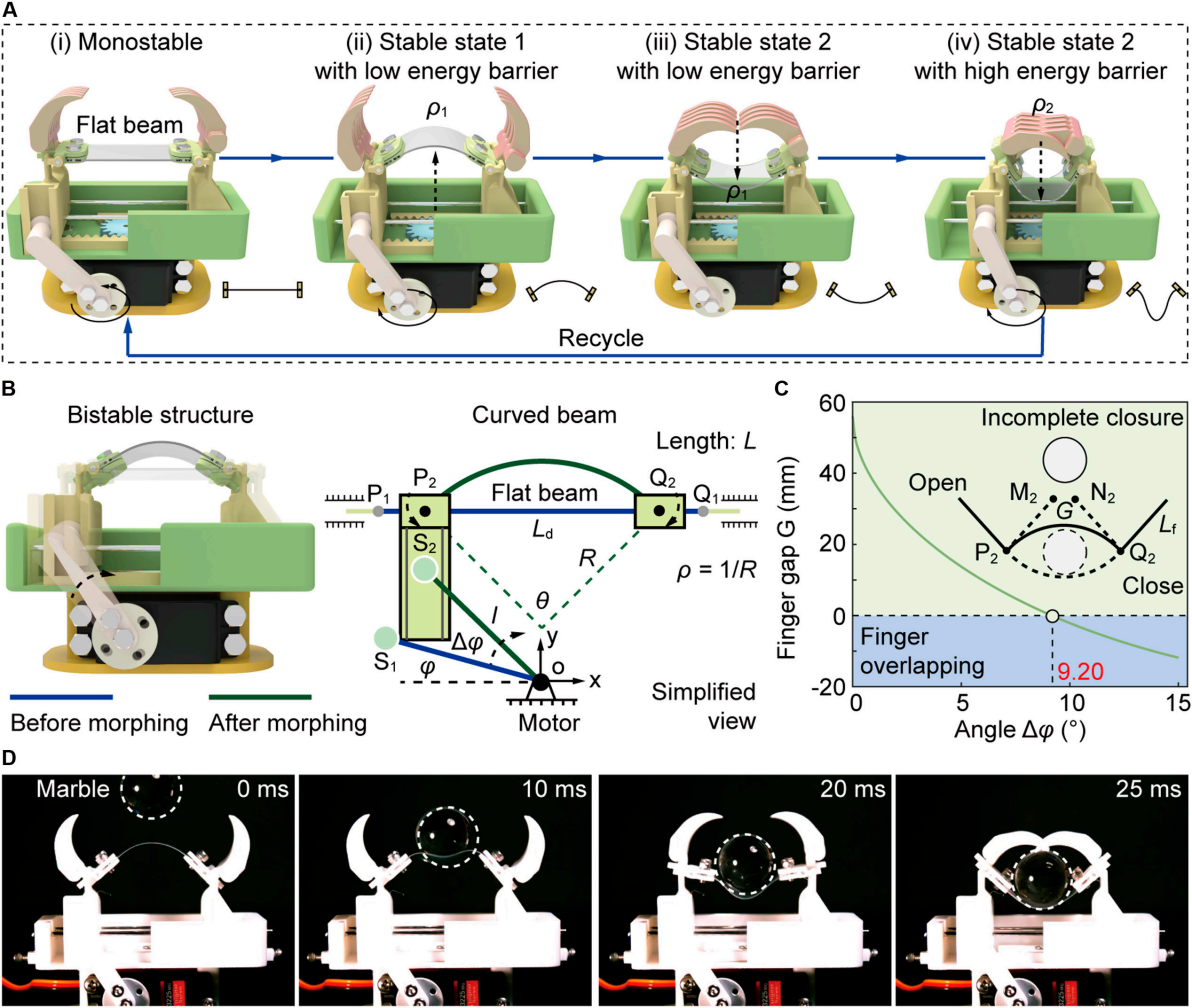
Operation principle and critical curvature of the robotic gripper. (A) Operational principle of the proposed robotic gripper. Leveraging the tunability of the energy barrier of the curved beam, our robotic gripper implements rapid, compliant, and powerful grasping behaviors. (B) Diagram of the bistable mechanism with tunable energy barriers. (C) Critical curvature of the initial configuration. Effects of the rotation angle Δ*φ* on the finger gap *G,* which is equal to the distance between points *M*_2_ and *N*_2_. (D) Robotic gripper rapidly transitioning the stable states under the action of a falling marble.

The operational principle of the robotic gripper is shown in Fig. 2A. Initially, the motor actuates the flat beam to experience bending deformation, transitioning it from a monostable to a bistable structure. Here, the beam bends upward with a curvature of *ρ*_1_, representing stable state 1 (energy barrier: Δ*E*_1_). In this state, the robotic gripper stays open with a low energy barrier, which enhances the sensitivity of robotic grippers and offers sufficient flexibility during contact with objects, minimizing the risk of damage. When the beam and objects are pressed against each other, the curved beam transitions from upward to downward bending, reaching stable state 2. During this transition, the clamps rotate, actuating the fingers to implement rapid grasping without the need for additional actuators. Although the fingers switch from an open to a closed state, the robotic gripper still maintains high sensitivity due to the consistent energy barrier of the beam. To further improve grasping stability, the motor is used to modify the curvature of the beam from *ρ*_1_ to *ρ*_2_ (energy barrier: Δ*E*_2_). This increased curvature significantly raises the energy barrier, thus enhancing the stability of the robotic gripper. By tuning the energy barrier in situ, the robotic gripper achieves rapid, compliant, and powerful grasping behavior. Moreover, this process is repeatable as the motor can continuously modulate curvature to accommodate different operational requirements.

### Critical curvature for achieving robotic gripper closure

Although switching between the stable states actuates the robotic gripper to grasp objects, insufficient initial curvature *ρ*_1_ will result in a gap between the fingers in stable state 2, causing objects to fall from the robotic gripper. To identify the critical curvature required for the fingers to close securely, a geometrical model is derived to analyze the influence of rotation angles Δ*φ* on the gap *G*, as demonstrated in Fig. 2B. To describe the position of these points, we build a coordinate system *xoy*, with the origin *o* located at the rotating shaft of the motor. In this model, *ζ*_1_ and *ζ*_2_ represent the position of point *ζ* before and after deformation, respectively. For example, the flat beam with a length of *L* is described as *P*_1_*Q*_1_, and the corresponding curved beam can be denoted as *P*_2_*Q*_2_, respectively. Moreover, the angle between the strut (length: *l*) and the negative direction of the *x* axis is expressed as *φ*. According to the geometrical relationship, when the strut rotates by Δ*φ*, the bending angle *θ* can be calculated (Supplementary Materials).

$$\theta =2\sqrt{1/{\left(1-2\left(l\text{cos}\varphi -l\text{cos}\left(\varphi +\Delta \varphi \right)\right)/L\right)}^{2}-1}$$ (1)

Subsequently, for the finger with a length of *L*_f_, the gap *G* between finger tips can be further calculated as

$$G=L_{d}-2L_{f}\text{sin}\left(\theta /2\right)$$ (2)

where *L*_d_ denotes the distance between 2 supports (i.e., *P*_2_*Q*_2_). In principle, *G* > 0 indicates that fingers fail to interlock when the robotic gripper is closed, increasing the risk of objects falling through. To achieve sufficient interlocking, a larger curvature *ρ*_1_ of the beam is required until *G* = 0. Consequently, the critical distance *L*_d_ for achieving the interlocking is determined to be 45.06 mm, as illustrated in Fig. 2C, corresponding to a rotation of the strut by 9.20° (Fig. S3A). Given the relationship between curvature and bending angle (*ρ* = *θ*/*L*), the resulting curvature *ρ*_1_ is calculated to be 0.04 mm^−1^, as shown in Fig. S3B. To validate this viewpoint, we fabricate a robotic gripper designed to meet these geometrical specifications, and the resulting grasping behavior is shown in Fig. 2D, where a marble falling from a height of 30 cm is applied to trigger the state transition. Upon impact, the beam is flattened within 10 ms (Movie S1). As the clamps rotate about their shafts, the beam transitions from an upward-bending to a downward-bending state. During this transition, the 2 fingers can interlock with each other, allowing the robotic gripper to grasp the marble within 25 ms (i.e., switching frequency: *f* = 40 Hz). Additionally, we also characterize the influence of diverse falling heights on response time, as shown in Fig. S4. When the robotic gripper successfully captures the marble, the gap *G* between the finger tips is measured as zero, verifying the accuracy of the theoretical model. Hence, when *L*_d_ is less than 45 mm, the gap between the 2 finger tips satisfies *G* < 0, preventing objects from falling.

### Compliant triggering of robotic gripper

**Fig. 3.**
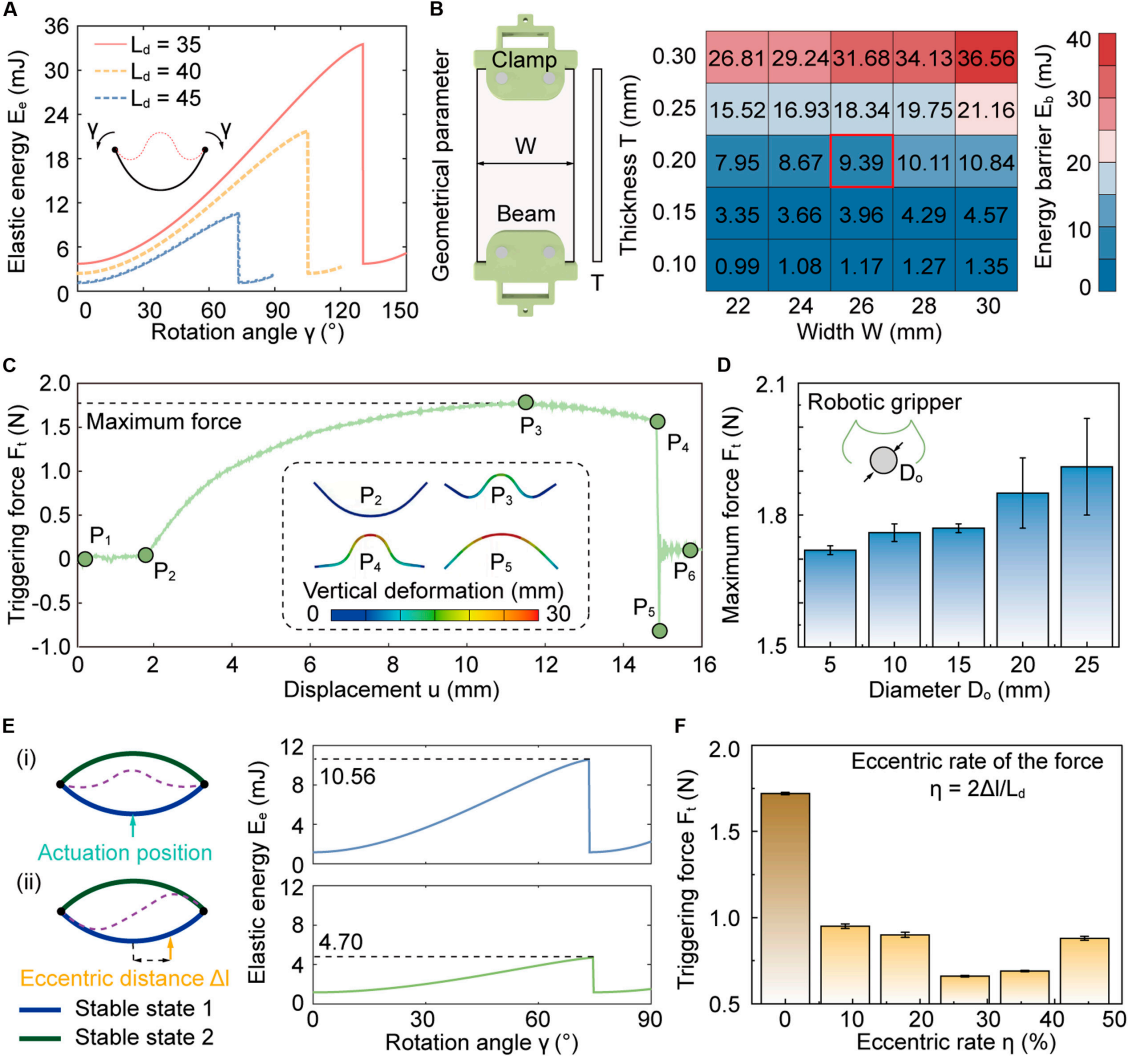
Compliant triggering of our robotic grippers. (A) Effects of rotation angle γ on elastic energy *E*_e_. (B) Relationship between geometric parameters, such as width *W* and thickness *T*, and energy barriers. (C) Force *F*_t_–displacement *u* curve of the curved beam during state transition. (D) Maximum triggering forces *F*_t_ of the robotic gripper during grasping cylindrical objects with a diameter of *D*_o_. (E) Comparison of energy barriers corresponding to the 2 diverse triggering approaches. (F) Relationship between maximum force *F*_t_ and eccentric rate *η*.

For bistable robotic grippers, the implementation of grasping behavior depends on the transition between stable states. Hence, minimizing the energy barrier essential for state transitions is a primary route to accomplish compliant triggering. To quantify the energy barrier of the beam, we construct a mechanical model capable of predicting its deformation by altering the rotation angle *γ* at its 2 ends (Supplementary Materials). As illustrated in Fig. S6 and Table S1, the consistency of the results obtained by finite element simulation verifies the effectiveness of the mechanical model. Employing this theoretical model, we further characterize the influence of distance *L*_d_, ranging from 35 to 45 mm with an increment of 5 mm, on the energy barrier, as depicted in Fig. 3A. First, changes in the distance *L*_d_ are uncovered to alter the initial curvature of the beam, which significantly impacts its elastic energy *E*_e_. As *L*_d_ declines from 45 to 35 mm, the curvature increases from 0.04 to 0.06 mm^−1^, which causes initial elastic energy *E*_1_ to rise by 3.18 times, reaching 3.72 mJ. Second, beams with higher curvature need greater rotational morphing to perform state transition. Meanwhile, as the angle *γ* increases, the elastic energy *E*_e_ will promptly accumulate, leading to a higher energy barrier. Among these cases examined, the energy barrier is minimized when *L*_d_ = 45 mm, at which the curved beam requires only 73.26° to initiate snap-through actions. By subtracting the initial energy *E*_1_ from the maximum elastic energy, the energy barrier for this profile is calculated to be only 9.39 mJ.

In addition to distance *L*_d_, geometrical parameters perform a critical role in altering the energy barrier. Considering that the structural length *L* of the beam remains invariable once the robotic gripper is fabricated, the analysis focuses on the effects of varying the width *W* and thickness *T*. As illustrated in Fig. 3B, while both parameters influence the energy barrier, the comparative results indicate that energy barrier is substantially more sensitive to variations in thickness *T* than in width *W*. Specifically, a reduction in thickness by merely 0.05 mm leads to a 57.86% decrease in the energy barrier, whereas declining the width by 2 mm results in a 7.69% reduction. Therefore, this indicates that altering thickness is, in principle, a more effective approach for promoting compliant triggering. However, excessively thin thickness will compromise the performance of grasping stability in turn. Therefore, to balance compliant triggering with powerful grasping, optimized geometrical parameters are determined with a width of 26 mm and a thickness of 0.20 mm for subsequent investigations (Fig. S2).

After identifying the critical parameters, we evaluate the triggering force *F*_t_ of the robotic gripper. Before experiments, we first mount the robotic gripper on the base of a testing machine. Subsequently, this robotic gripper is applied to grasp a cylindrical stick with a diameter *D*_o_ of 5 mm (Fig. S7 and Movie S2). Here, as the contact position coincides with the center line of the curved beam, this process is named central triggering. Under this quasi-static compression, the force–displacement (i.e., *F*_t_–*u*) curve is demonstrated in Fig. 3C. To illustrate the experimental curve, we define 6 critical points *P*_i_ (*i* = 1, …, 6) to segment it into distinct sections. In the initial phase (*P*_1_ to *P*_2_), prior to contact between the beam and the stick, the force *F*_t_ remains zero. As the beam begins to compress the stick (*P*_2_ to *P*_3_), the force *F*_t_ increases gradually from zero to a maximum value of 1.72 N at a displacement of 11.78 mm. Subsequently, the beam transitions into a negative stiffness regime (*P*_3_ to *P*_4_). The snap-through phenomenon does not occur immediately since the energy required for triggering has not yet been fully accumulated. Then, when the displacement *u* reaches 14.83 mm, the snap-through is triggered, causing the beam to transition to stable state 2 (*P*_4_ to *P*_5_). During this transition, the force *F*_t_ immediately decreases from 1.61 to −0.82 N. Following the snap-through, compression between the beam and the stick is lost, resulting in the force *F*_t_ returning to zero (*P*_5_ to *P*_6_). During this process, the local deformation of the curved beam is also showcased in Fig. 3C. In addition, the force–displacement curves for sticks with varying diameters, ranging from 5 to 25 mm, are characterized. Across these cases, the maximum force *F*_t_ generated by compressing the center of the beam showcases an upward trend with the increase of diameter *D*_o_, from 1.72 to 1.91 N, as shown in Fig. 3D.

Compared to compressing the center of the curved beam, the state transition becomes easier when the actuation position deviates from the center of the beam, i.e., eccentric triggering. Here, the eccentric distance is defined as Δ*l*. To theoretically explain this reason, we analyze the variations in the elastic energy associated with the 2 triggering approaches employing the mechanical model, as shown in Fig. 3E. The results indicate that the maximum elastic energy *E*_e_ required for eccentric triggering is 4.70 mJ, which is only half of that required for central triggering. The significant reduction in the energy barrier is the primary reason for the facilitated state transition. To further investigate the influence of eccentric distance Δ*l* on triggering force, we introduce the eccentric rate, denoted as *η* = 2Δ*l*/*L*_d_, and measure the force *F*_t_ at diverse eccentric positions, aiming to identify the optimal eccentric rate, as demonstrated in Fig. 3F. The results reveal 2 critical findings. On the one hand, across all eccentric rate, the triggering force remains significantly lower than that generated by central triggering, consistently staying below 1 N, which indicates that eccentric triggering enables a more compliant interaction with environments. On the other hand, when the rate *η* reaches 26.67% (i.e., Δ*l* = 6 mm), the force *F*_t_ reaches its minimum value of only 0.66 N, highlighting a critical threshold for optimal triggering efficiency. These insights highlight the effectiveness of eccentric triggering strategy in lowering energy barriers and fine-tuning the mechanical response of the bistable structures, thereby enhancing the adaptability of the robotic gripper. Here, we also emphasize that this triggering force has no significant influence on the shape and material properties of the object, as shown in Fig. S8. The reduced energy barriers enhance triggering flexibility, enabling the robotic gripper to transition seamlessly from an open to a closed profile upon rapid contact with floating objects (Fig. S9 and Movie S4). Moreover, the robotic gripper maintains its stable configuration during high-speed (1.80 m/s) and high-acceleration (3.0 m/s^2^) approaches, demonstrating strong resistance to unintended activation and confirming its robust anti-interference performance.

### Powerful grasping of robotic gripper

**Fig. 4.**
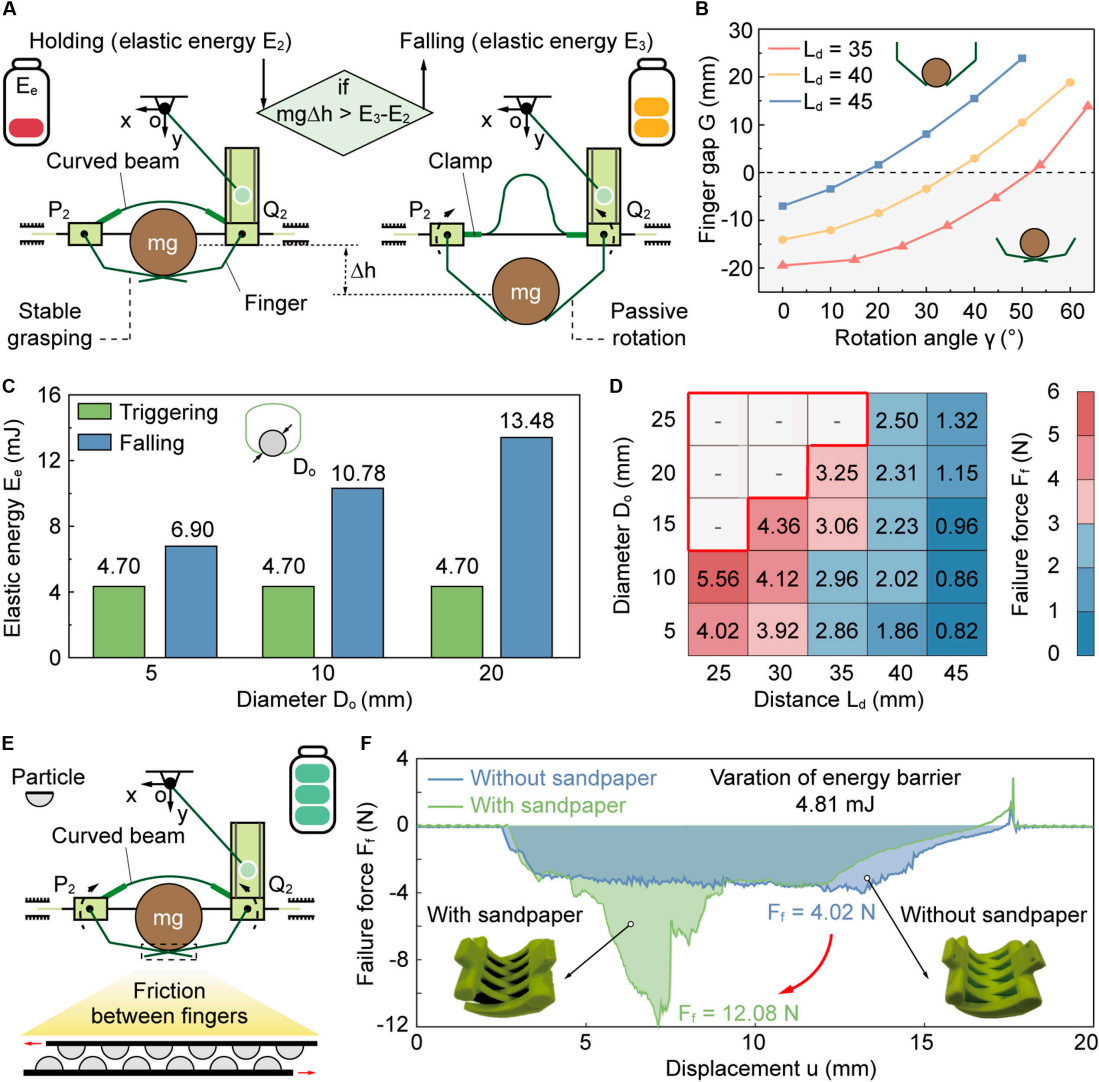
Powerful grasping of our robotic gripper. (A) Schematic diagram of the object falling from the robotic gripper. *E*_2_ and *E*_3_ represent the elastic energy of the curved beam during holding and falling, respectively. (B) Gap *G* caused by the rotation of the fingers. A negative gap between the finger tips indicates that they intersect with each other. (C) Comparison of elastic energy of the curve beam during triggering and falling. (D) Effects of both distance *L*_d_ and diameter *D*_o_ on the failure force *F*_f_. The red box represents the situation where the diameter of the object exceeds the grasping range of the robotic gripper. (E) By leveraging friction between the fingers to alter energy barrier for increasing the failure force *F*_f_. (F) Force *F*_f_–displacement *u* curve of the robotic gripper during object detaching.

After executing the grasping action, we also analyze the failure mechanism of the robotic gripper, as shown in Fig. 4A. Initially, when the robotic gripper securely holds on a cylindrical stick with a mass of *m*, the elastic energy stored in the curved beam is denoted as *E*_2_. Here, this elastic energy provides the primary resistance against external disturbances and maintains a powerful grasping. Subsequently, if the gravitational force exerted by an object exceeds the load-bearing capability of the robotic gripper, it causes the fingers to passively rotate outward by an angle of *γ* around rotating shafts. Assuming negligible energy dissipation in the robotic system, the gravitational potential energy is converted into the elastic energy of the curved beam, increasing it to *E*_3_. The grasping stability is determined by the balance between the energy variations. Specifically, when the increase in elastic energy (i.e., *E*_3_–*E*_2_) due to beam deformation is sufficient to offset the gravitational potential energy, the object remains securely grasped. On the contrary, if the gravitational potential energy surpasses the additional elastic energy stored in the beam, the object will experience continuous downward sliding. Ultimately, the grasping failure occurs when the diameter *D*_o_ is less than the gap *G* between the fingers, causing the stick to fall from the robotic gripper.

Building upon the aforementioned analysis, we identify a fundamental distinction between the failure and trigger mechanisms of the robotic gripper. Specifically, different from the trigger mechanism, which involves a transition between stable states via snap-through instability, grasping failure occurs when the gap between the finger tips exceeds the diameter of objects, leading to an unintended release. Here, due to the lack of energy barrier required for transitioning between stable states, the force required for the stick to detach, i.e., failure force *F*_f_, is always lower than the corresponding triggering force *F*_t_. This discrepancy introduces a trade-off between compliant triggering and powerful grasping, presenting an inherent challenge in the design of bistable robotic grippers. To mitigate this limitation, we attempt to improve the grasping stability by increasing the rotation angle *γ* necessary for stick detachment. This allows our bistable robotic gripper to securely hold objects under external disturbances, thereby reducing the likelihood of unintended release, as illustrated in Fig. 4B. Specifically, when *L*_d_ is set to 45 mm, the curved beam only needs to rotate 30° for an object with a diameter of 10 mm to detach. By contrast, the angle *γ* increases to 50° after reducing *L*_d_ to 40 mm, strengthening the ability to hold the object. This increase in the necessary angle implies a higher energy threshold that must be overcome for detachment, thus enhancing grasping stability. It is worth noting that the value of the energy threshold is determined by the energy barrier of the system. Furthermore, even when *L*_d_ remains constant, the angle *γ* for detachment varies depending on the diameter of the object being grasped. For example, when *L*_d_ is arranged to 40 mm, an object with a diameter of 10 mm requires a rotation of 50° to detach, while the other object with a large diameter of 20 mm necessitates an additional 10° of rotation before it falls from the robotic gripper. This extra angular displacement corresponds to an additional 2.70 mJ of elastic energy *E*_e_ that needs be overcome, making detachment significantly more difficult, as shown in Fig. 4C. This internal mechanism directly impacts the force *F*_f_ of the robotic gripper, as the amount of energy required for object detachment dictates its grasping stability. To further quantitatively evaluate this effect, we evaluate the force *F*_f_ under varying conditions, with the results presented in Fig. 4D and Fig. S6 (Movie S3). Our findings reveal that tuning *L*_d_ (from 45 to 25 mm) significantly enhances the grasping performance of the robotic gripper, which allows the maximum force *F*_f_ to reach 5.56 N. By contrast, the force *F*_f_ is substantially lower for the robotic gripper with *L*_d_ = 45 mm, being merely 1.32 N. This comparison highlights the critical role of energy barrier tuning in enhancing grasping stability.

Beyond altering the curvature of the beam, the energy barrier of the robotic system can be further modulated by introducing friction between the fingers (Fig. 4E). Notably, friction does not interfere with the initial triggering grasp, but instead serves to increase the failure force *F*_f_, without compromising the compliant nature of the grasp. To analyze the effect of friction on grasping stability, sandpaper is applied to the contact points of 2 fingers. Subsequently, the corresponding force *F*_f_, required to detach an object from the robotic gripper, is evaluated (Fig. S10). As depicted in Fig. 4F, a 5-mm stick requires overcoming an additional energy barrier of 4.81 mJ due to friction to be released from the robotic gripper. The friction-induced modification results in a 3-fold increase in the maximum force *F*_f_ compared to a frictionless robotic gripper. The increase in force *F*_f_, caused by the enhanced energy barrier, significantly contributes to the stability of the grasp. Although friction improves failure force, excessive friction may impair the ability to reset. Here, due to the actuation torque limitations of the motor, the maximum achievable failure force through this method is estimated to be approximately 12 N.

### Automatically tuning energy barrier during manipulation

**Fig. 5.**
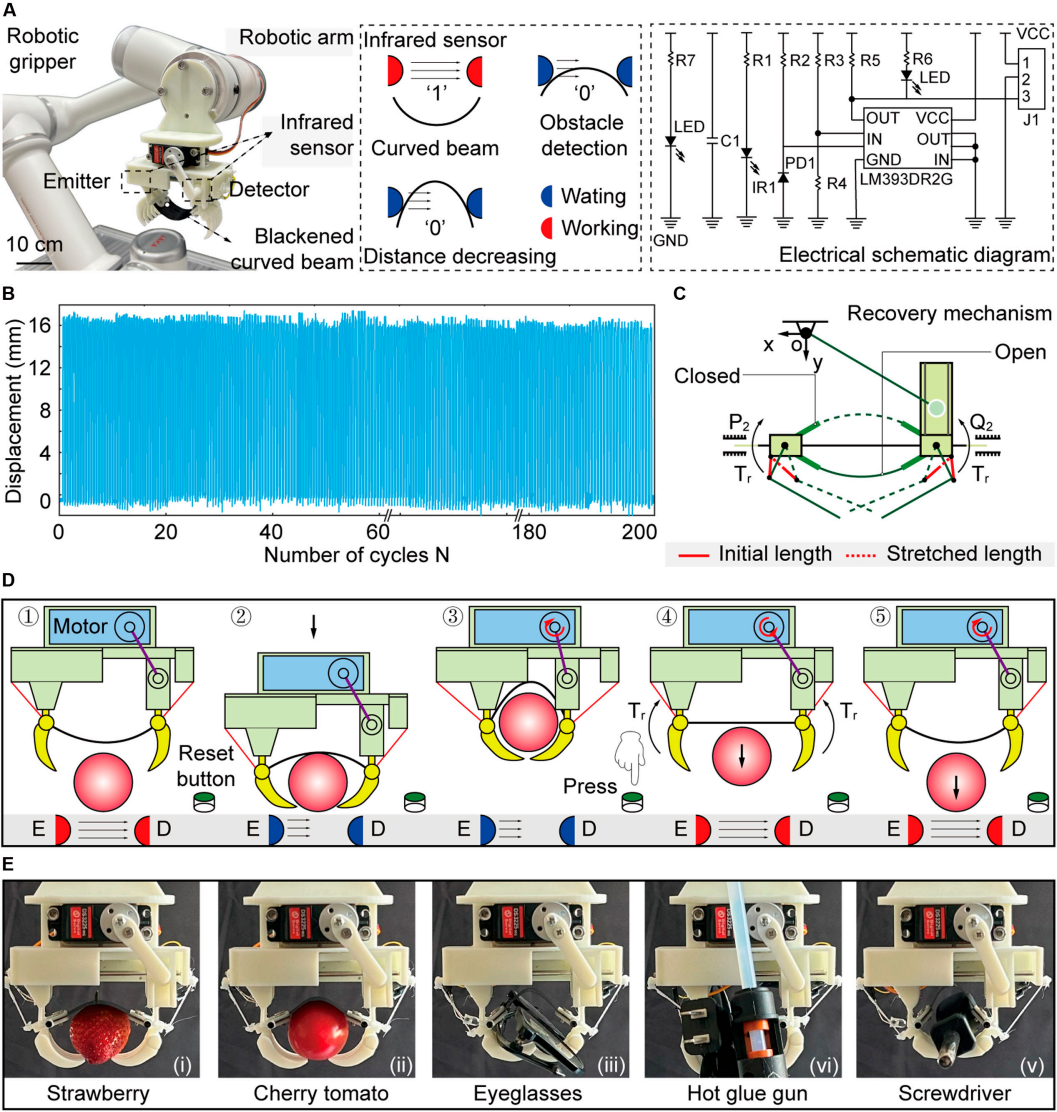
Application demonstration of our bistable robotic gripper. (A) Principle of automatic regulation of energy barriers by applying infrared sensors. Here, the state is designated as “1” when the signal from the emitter is detected; otherwise, it is designated as “0”. (B) Durability evaluation of curved beams by cycle testing. (C) Recovery mechanism of the robotic gripper. (D) Automated process for grasping and releasing items. (E) Robotic gripper performing compliant and adaptive grasping behavior.

To enhance the functionality of bistable robotic grippers in practical applications, we first mount it onto a rigid robotic arm (C5, JAKA, China), as illustrated in Fig. 5A. Subsequently, infrared sensors are installed on both supports to facilitate autonomous sensing of state transitions. When the beam remains in stable state 1, the infrared signal emitted from a light-emitting diode (i.e., emitter) is detected by the other one (i.e., detector), which indicates that the robotic gripper is in an open state. Upon contact with an object, the beam transitions to stable state 2, causing the infrared signal to be blocked, at which the signal changes from 1 to 0. By using this signal as input, the motor can be driven to increase the energy barrier. Here, the duration of energy barrier modulation is ~300 ms (Note S4 and Movie S5). To ensure effective signal blockage, the transparent curved beam is coated with carbon black, thereby enhancing infrared absorption and minimizing interference from ambient light. We conduct over 200 cycles of the bending process of the robotic gripper to evaluate the durability of the curved beam (Fig. 5B and Fig. S12). Then, 2 elastic cables (highlighted in red in Fig. 5C) are strategically positioned between the supports and the clamps. The cables generate elastic restoring forces that provide the necessary torque *T*_r_ to ensure that the robotic gripper is reset to the open state after releasing objects. The reset function makes robotic grippers perform continuously operational efficiency.

After integrating sensors into the design of the robotic gripper, we demonstrate the automated functionality of the robotic gripper, achieving rapid, compliant, and powerful grasping. The regulation of beam curvature, which governs the grasping and releasing process, is entirely controlled by motor actuation. Furthermore, the reset of the robotic gripper is performed by pressing a button, streamlining the operation, as shown in Fig. 5D (Movie S6). Based on the described operational principles, the robotic gripper shows its capability to adaptively handle a diverse range of objects with varying shapes, sizes, and material properties (Fig. S13). As shown in Fig. 5E, the robotic gripper manipulates objects, for example a delicate strawberry, a rigid screwdriver, and a pair of eyeglasses, which highlights its versatility in handling items with different mechanical properties.

Moreover, we conduct a comparative experiment to validate the effect of energy barrier modulation on carrying capacity (Fig. S14 and Movie S7). The experimental results illustrate that the robotic gripper showcases a significant improvement in load-bearing capacity when the energy barrier is elevated. Specifically, the robotic gripper picks up an object weighing 500 g successfully by raising the curvature of the beam, indicating its capability to maintain a powerful grasp under higher energy conditions. In contrast, if the robotic gripper manipulates at a low energy barrier, the grasping force generated is insufficient to counteract the weight. The beam undergoes excessive deformation, compromising the stability of bistable robotic grippers. This deformation ultimately leads to the unintended release of the object, which falls through the gap between the 2 finger tips, resulting in grasp failure. These findings underscore the adaptability of our robotic gripper, showcasing its capability to dynamically program its energy barrier to accommodate varying load requirements. By achieving a balance between compliant and powerful grasping, the robotic gripper can effectively transition between compliant manipulating of delicate items and powerful grasping of heavier items. This customized grasping strategy enhances the versatility and reliability of the robotic gripper, making it a promising solution for applications requiring both precision and strength in robotic manipulation.

### UAV perching enabled by our robotic gripper

**Fig. 6.**
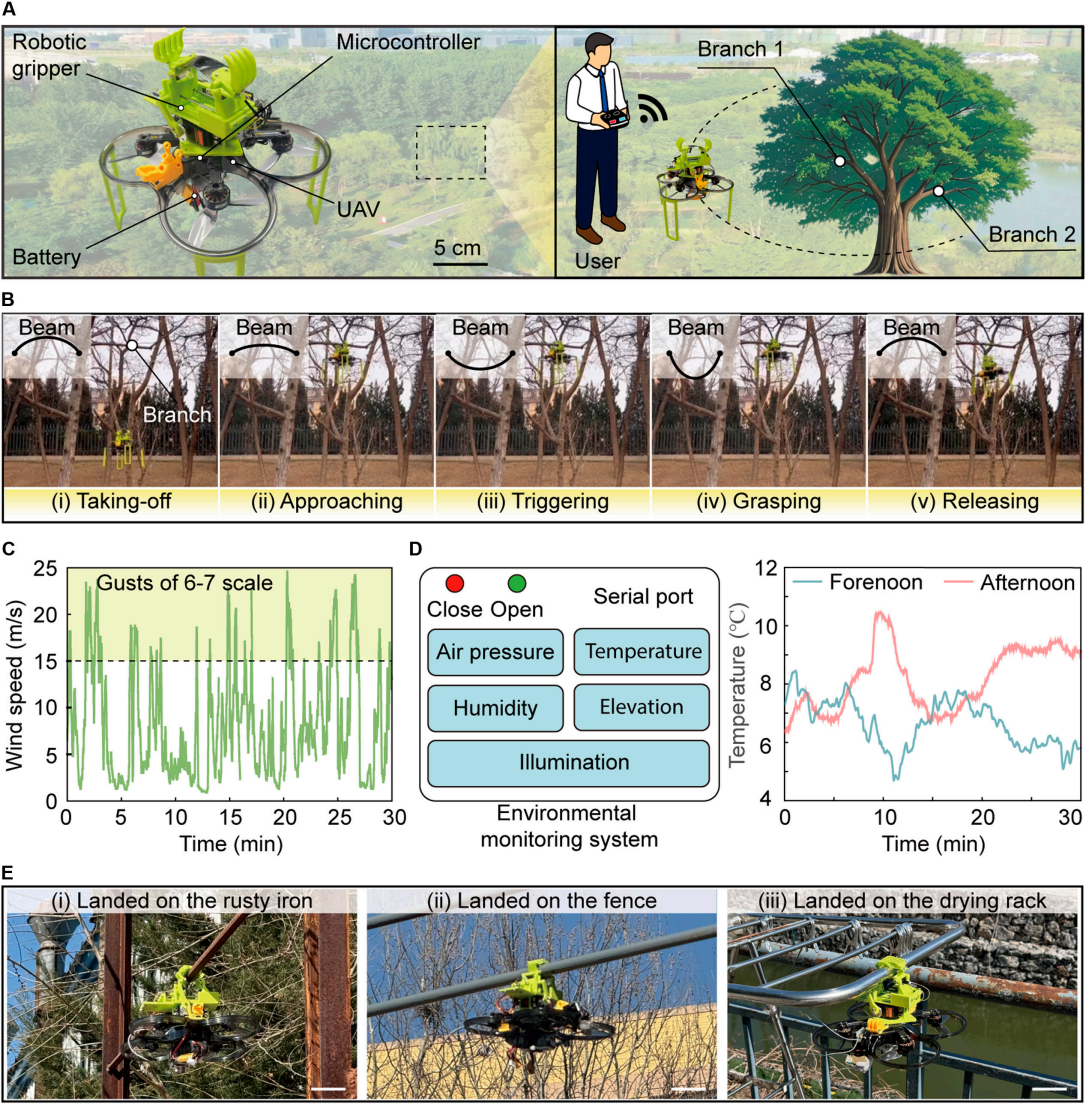
UAV perching enabled by our robotic gripper. (A) A UAV equipped with our robotic gripper. (B) UAV perches without consuming energy by grasping branches. (C) Variation of wind speed during the UAV perching. (D) Environmental monitoring such as temperature and humidity utilizing sensors on the UAV. (E) Robotic gripper-assisted UAVs perch on diverse environments. Scale bar, 10 cm.

Bistable robotic grippers, traditionally engineered for grasping applications, have exhibited significant potential in assisting UAVs in perching tasks, as shown in Fig. 6A. In contrast to traditional robotic grippers, which require precise coordination between the UAV’s flight dynamics and robotic gripper actuation, bistable grippers can leverage a passive triggering mechanism that allows automatic engagement upon contact. This passive response markedly reduces the dependence on fine maneuvering and complex control algorithms, thus improving the reliability and operational robustness of UAV perching, particularly in unpredictable environments. However, achieving a balance between compliant triggering and powerful perching presents a crucial challenge. A low energy barrier facilitates more compliant triggering, allowing the system to engage with perching surfaces employing minimal external force. However, this advantage comes at the cost of reduced stability [28]. When applied in perching scenarios, such as landing on tree branches, these robotic grippers may render UAVs more susceptible to external disturbances, increasing the risk of detachment or failure, such as falling from the branches. To enhance grasping stability, conventional approaches involve increasing the energy barrier of the bistable structures [47]. While effective in strengthening attachment, this approach necessitates UAVs to exert a greater impact force during perching to transition between stable states. This increased force not only raises energy consumption but also heightens the risk of damage to sensitive electronic components, limiting the system’s long-term reliability. Therefore, the ability to on-demand switch between flexibility for triggering and stability for perching is essential for optimizing UAV performance in practical applications.

The integration of our robotic gripper into UAV development effectively addresses this limitation, as illustrated in Fig. 6B. The system enables UAVs to interact with branches applying the robotic gripper that operates with minimal energy consumption (Movie S8). Upon initiating a stable state transition, the robotic gripper instantaneously increases its energy barriers through motor actuation, ensuring a secure attachment. Notably, once these energy barriers are activated, the UAV remains firmly fixed to the branch, even in the absence of an external power supply, maintaining stability without continuous energy input. To assess perching stability, a wind speed sensor is incorporated into the UAV, allowing real-time evaluation of its anchoring performance under varying environmental conditions. As shown in Fig. 6C, the system successfully maintains its grasp on branches even under wind gusts of 6 to 7 scale. When the task is completed, the robotic gripper can be remotely disengaged via Bluetooth, releasing the UAV from the branch. Meanwhile, the robotic gripper autonomously reverts to its initial stable state, ensuring rapid readiness for the subsequent tasks. This self-resetting ability enhances operational efficiency by eliminating the requirement for manual intervention. Beyond perching, the integration of this robotic gripper significantly broadens the functional scope of the UAV across diverse applications. For example, when stationed on a tree branch, the system serves as a stable platform for deploying onboard sensors, enabling continuous environmental monitoring. This ability facilitates real-time data acquisition on critical parameters such as temperature and humidity (Fig. 6D). Such data collection is particularly valuable for ecological research, climate monitoring, and precision agriculture, where localized environmental insight is essential. In addition to perching on branches, the robotic gripper enables our UAV to anchor onto various surfaces and objects, as shown in Fig. 6E. This adaptability extends its operational scope to urban environments and artificial structures. Moreover, UAVs equipped with lightweight solar panels can leverage our robotic gripper to establish stable perching position in sunlit locations, enabling in situ energy harvesting. This capability may provide a sustainable energy recovery approach, allowing UAVs to extend their operational duration without frequent battery replacements or external charging stations. Such advancements are beneficial for applications requiring persistent surveillance, disaster response, and long-term remote sensing missions.

## Discussion

Bistable structures have been widely utilized in developing robotic systems due to their unique advantages, including rapid response, high-output force, and power amplification. However, existing bistable structures typically feature invariable energy barriers, limiting their adaptability to varying demands. In this combining theoretical and experimental study, we present a bistable robotic gripper capable of dynamically modulating its energy barrier by altering the curvature of an elastic beam. This tunability enables precise control over the balance between compliant triggering and powerful grasping. In its low-energy barrier state, the robotic gripper exhibits high compliance, requiring merely 0.66 N of triggering force to initiate grasping, making it particularly suitable for handling fragile objects. Once the grasp is established, increasing the energy barrier significantly enhances stability, accomplishing a failure force of 12.08 N. This tunable energy barrier mechanism results in an exceptional failure-to-triggering ratio of 18.30, demonstrating its effectiveness in optimizing sensitivity and grasping strength (Table S2).

Over the past decade, researchers have developed diverse bistable structures that operate without actuators, utilizing inherent mechanical instabilities to enable passive grasping [25,48,49]. These designs provide advantages such as simplicity, lightweight construction, and energy efficiency. However, their inability to modulate energy barriers results in low failure -to-triggering force ratios, typically below 0.50, limiting their effectiveness in applications requiring gentle handling of delicate objects and firm holding of heavier loads. To overcome this limitation, recent advancements have proposed actuation strategies that enable dynamic energy barrier modulation in robotic grippers [50,51]. By actively programming the energy landscape, these robotic grippers enhance grasping stability by allowing the failure force to exceed the triggering force (i.e., κ > 1). Despite these improvements, existing bistable robotic grippers struggle to achieve an optimal balance between compliant triggering and powerful grasping. Compliant triggering reduces the required force for handling fragile objects, while strong grasping ensures secure retention under external disturbances—a critical trade-off for practical applications. Beyond functional challenges, many bistable robotic grippers rely on complex mechanisms or multiple actuators, which introduce additional weight, increase fabrication complexity, and reduce system reliability. These drawbacks restrict their widespread adoption, particularly in mobile robotic systems where both lightweight design and high-performance grasping are essential.

Our bistable robotic gripper addresses these challenges by utilizing a simple yet effective mechanism to alter energy barriers of an elastic curved beam, enabling real-time modulation of grasping behavior. This design maintains structural simplicity while achieving a greatly improved failure-to-triggering force ratio. By dynamically programming the energy barrier, robotic grippers seamlessly transition between compliant triggering and powerful grasping, adapting to various manipulation needs. To showcase its practical application, we integrate robotic grippers into UAVs, enabling stable perching on different surfaces. This integration highlights its ability to facilitate energy-efficient and adaptive grasping, enhancing the functionality of aerial robotics. With its rapid response, programmable interaction forces, and mechanically efficient design, our robotic gripper presents a promising solution for next-generation robotic systems. In addition to robotic grippers, introducing this modulation strategy for developing a variety of bistable structures opens possibilities for attracting more advanced technologies into robotic applications [52,53]. Even so, we acknowledge that the current prototype, which depends on a motor to dynamically program the energy barrier, may pose limitations for robotic applications, especially those requiring ultra-lightweight designs. Therefore, future work will focus on optimizing the system by incorporating smart actuators based on responsive materials as an alternative to motors [54,55]. The materials have the significant potential to reduce weight while enhancing adaptability, responsiveness, and energy efficiency. In addition to weight reduction, we will also use 3D printing technology to develop diverse bistable structures, and strive to explore the effects of the configuration selection on the performance of robotic systems [56–58].

## Materials and Methods

### Fabrication of robotic gripper

In this robotic gripper, the curved beam was made of polyethylene terephthalate (PET) with a thickness *T* of 0.20 mm (length *L* = 50 mm and width *W* = 26 mm). Theoretically, the energy barrier could be programmed broadly through the change in the material and thickness of the beam. To alter the curvature of this beam, we applied a slider-crank mechanism that was actuated by a steering engine (DS3120, DSSERVO, China). The working torque of this steering engine with a mass of 60 g was 15 kg/cm. Here, curved beams, polishing rods, and steering engine were commercially available components. The remaining components were 3D-printed using photosensitive resin (SOMOS Imagine 80000/SOMOS GP Plus).

### Capturing rapid grasping behavior

We applied a high-speed camera (Phantom, VEO-E 310L, USA), equipped with micro-lens (Canon, EF100mmf/2.8LISUSM, Japan), to record the state transition of the robotic gripper. To capture the transient state transition, the sampling frequency of the camera was set to 500 frames per second.

### Measurement of quasi-static mechanical behavior

The quasi-static mechanical response of the curved beam was obtained applying a uniaxial tensile testing machine (ESM303, Mark-10, USA). In this machine, the force sensor has a capacity of 10 N and the corresponding resolution reaches 0.01 N. During conducting experiments, the robotic gripper was first mounted on the machine and then moved vertically to contact cylindrical sticks with a velocity of 60 mm/min. Using this machine, the force–displacement curve could be obtained.

### Assembly of robotic gripper-enabled UAVs

The UAV (MVD35, MicroAir, China) was commercially available and controlled by a Pixhawk flight microcontroller. It was equipped with a 1500 mAh lithium battery (CD6S1500120HV, CODDAR, China) mounted on bottom, providing sufficient power for 13 min of flight time. During approaching branches, an optical flow ranging sensor (MTP-01P, MicroAir, China) was used to maintain flight height, enhancing the controllability of the system. To save energy when flight was not required, the bistable robotic gripper was installed at the top of the UAV. Here, the weight of this system was evaluated as 548.10 g.

## Data Availability

The authors declare that the main data supporting the findings of this study are available within the article.

## References

[1] Zhou L, Ren L, Chen Y, Niu S, Han Z, Ren L. Bio-inspired soft grippers based on impactive gripping. Adv Sci. 2021;8(9):2002017. 10.1002/advs.202002017 PMC8097330 33977041

[2] Tai K, El-Sayed A-R, Shahriari M, Biglarbegian M, Mahmud S. State of the art robotic grippers and applications. Robotics. 2016;5(2):11.

[3] Birglen L, Schlicht T. A statistical review of industrial robotic grippers. Robot Comput Integr Manuf. 2018;49:88–97.

[4] Wu M, Afridi WH, Wu J, Afridi RH, Wang K, Zheng X, Wang C, Xie G. Octopus-inspired underwater soft robotic gripper with crawling and swimming capabilities. Research. 2024;7:0456. 39206446 10.34133/research.0456 PMC11350063

[5] Zhang J, Shi J, Zhao Y, Yang J, Rajabi H, Peng H, Wu J. Bio-inspired tensegrity building block with anisotropic stiffness for soft robots. IEEE/ASME Trans Mechatron. 2025;1–12.

[6] Li F, Yang H, Gu G, Wang Y, Peng H. Position and orientation tracking control of a cable-driven tensegrity continuum robot. IEEE Trans Robot. 2025;41:1791–1811.

[7] Hao Y, Gong Z, Xie Z, Guan S, Yang X, Wang T, Wen L. A soft bionic gripper with variable effective length. J Bio Eng. 2018;15:220–235.

[8] Zhao Y, Zhang J, Zhang S, Zhang P, Dong G, Wu J, Zhang J. Transporting dispersed cylindrical granules: An intelligent strategy inspired by an elephant trunk. Adv Intell Syst. 2023;5:2300182.

[9] Ke X, Jang J, Chai Z, Yong H, Zhu J, Chen H, Guo CF, Ding H, Wu Z. Stiffness preprogrammable soft bending pneumatic actuators for high-efficient, conformal operation. Soft Robot. 2022;9(3):613–624. 34255577 10.1089/soro.2020.0207

[10] Junfeng H, Guilin W, Jie L, Liang X, Xie YM. A modular continuous robot constructed by Miura-derived origami tubes. Int J Mech Sci. 2024;261:108690.

[11] Liu J, Chen Z, Wen G, He J, Wang H, Xue L, Long K, Xie YM. Origami chomper-based flexible gripper with superior gripping performances. Adv Intell Syst. 2023;5:2300238.

[12] Firouzeh A, Paik J. An under-actuated origami gripper with adjustable stiffness joints for multiple grasp modes. Smart Mater Struct. 2017;26:055035.

[13] Hong Y, Zhao Y, Berman J, Chi Y, Li Y, Huang H, Yin J. Angle-programmed tendril-like trajectories enable a multifunctional gripper with ultradelicacy, ultrastrength, and ultraprecision. Nat Commun. 2023;14(1):4625. 37532733 10.1038/s41467-023-39741-6 PMC10397260

[14] Yang Y, Vella K, Holmes DP. Grasping with kirigami shells. Sci Robot. 2021;6(54): Article eabd6426. 34043535 10.1126/scirobotics.abd6426

[15] Guo J, Li Z, Low J-H, Han Q, Chen C-Y, Liu J, Liu Z, Yeow C-H. Kirigami-inspired 3D printable soft pneumatic actuators with multiple deformation modes for soft robotic applications. Soft Robot. 2023;10(4):737–748. 36827310 10.1089/soro.2021.0199

[16] Shintake J, Cacucciolo V, Floreano D, Shea H. Soft robotic grippers. Adv Mater. 2018;30:1707035. 10.1002/adma.201707035 29736928

[17] Jiang Y, Tong X, Li J, Li H, Cao C, Gao X, Li Y. Reprogrammable bistable actuators for multimodal, fast, and ultrasensitive grasping. IEEE/ASME Trans Mechatron. 2023;29(2):984–994.

[18] Chi Y, Li Y, Zhao Y, Hong Y, Tang Y, Yin J. Bistable and multistable actuators for soft robots: Structures, materials, and functionalities. Adv Mater. 2022;34(19):2110384. 10.1002/adma.202110384 35172026

[19] Cao Y, Derakhshani M, Fang Y, Huang G, Cao C. Bistable structures for advanced functional systems. Adv Funct Mater. 2021;31(45):2106231.

[20] Albertini F, Tarantino MG, Daniel L. Mechanical behavior of embedded bistable dome shell with tunable energy barrier asymmetry. Int J Mech Sci. 2024;263:108762.

[21] Yang H, Zhang J, Wang J, Hu J, Wu Z, Pan F, Wu J. Delocalized deformation enhanced reusable energy absorption metamaterials based on bistable tensegrity. Adv Funct Mater. 2025;35(5):2410217.

[22] Zhang P, Tang B. A two-finger soft gripper based on bistable mechanism. IEEE Robot Autom Lett. 2022;7(4):11330–11337.

[23] Hou N, Wu M, Zhao Q, Tang Z, Wang K, Xu X, Zheng X, Xie G. Reticular origami soft robotic gripper for shape-adaptive and bistable rapid grasping. Soft Robot. 2024;11(4):550–560. 39178400 10.1089/soro.2023.0051

[24] Mungekar M, Ma L, Yan W, Kackar V, Shokrzadeh S, Jawed MK. Design of bistable soft deployable structures via a kirigami-inspired planar fabrication approach. Adv Mater Technol. 2023;8(4):2300088.

[25] Zhang Y, Quan J, Li P, Song W, Zhang G, Li L, Zhou D. A flytrap-inspired bistable origami-based gripper for rapid active debris removal. Adv Intell Syst. 2023;5(7):2200468.

[26] Zhang X, Wang Y, Tian Z, Samri M, Moh K, McMeeking RM, Hensel R, Arzt E. A bioinspired snap-through metastructure for manipulating micro-objects. Sci Adv. 2022;8(46): Article eadd4768. 36399572 10.1126/sciadv.add4768 PMC9674295

[27] Hua J, Zhou Y, Chen CQ. Design and analysis of a tunable multistable mechanical metamaterial. Int J Mech Sci. 2024;272:109170.

[28] Zhang H, Lerner E, Cheng B, Zhao J. Compliant bistable grippers enable passive perching for micro aerial vehicles. IEEE/ASME Trans Mechatron. 2020;26(5):2316–2326.

[29] Zhang H, Sun J, Zhao J. Compliant bistable gripper for aerial perching and grasping. In: *2019 International Conference on Robotics and Automation (ICRA)*. Montreal (QC, Canada): IEEE; 2019. p. 1248–1253.

[30] Pal A, Goswami D, Martinez RV. Elastic energy storage enables rapid and programmable actuation in soft machines. Adv Funct Mater. 2020;30:1906603.

[31] Tang Y, Chi Y, Sun J, Huang T-H, Maghsoudi OH, Spence A, Zhao J, Su H, Yin J. Leveraging elastic instabilities for amplified performance: Spine-inspired high-speed and high-force soft robots. Sci Adv. 2020;6: Article eaaz6912. 32494714 10.1126/sciadv.aaz6912 PMC7209986

[32] Chi Y, Hong Y, Zhao Y, Li Y, Yin J. Snapping for high-speed and high-efficient butterfly stroke–like soft swimmer. Sci Adv. 2022;8: Article eadd3788. 36399554 10.1126/sciadv.add3788 PMC9674291

[33] Chen T, Bilal OR, Shea K, Daraio C. Harnessing bistability for directional propulsion of soft, untethered robots. Proc Natl Acad Sci USA. 2018;115(22):5698–5702. 29765000 10.1073/pnas.1800386115 PMC5984517

[34] Bambrick T, Viquerat A, Siddall R. Does bistability improve swimming performance in robotic fish? Adv Intell Syst. 2024;6(6):2300748.

[35] Jiang L, Li B, Ma F, Chen G. A tristable actuator for a bidirectional crawling and falling-rebootable robot. IEEE/ASME Trans Mechatron. 2023.

[36] Zhang Z, Nan R, Shen H, Pan B, Zhang G, Sun M, Chai H, Jiang S. A high load capacity and efficient-transporting inchworm-like crawling robot with bistable structure and pneumatic networks actuator. Smart Mater Struct. 2023;32(12):125009.

[37] Drotman D, Jadhav S, Sharp D, Chan C, Tolley MT. Electronics-free pneumatic circuits for controlling soft-legged robots. Sci Robot. 2021;6(51): Article eaay2627. 34043527 10.1126/scirobotics.aay2627

[38] Guo Q, Sun Y, Zhang T, Xie S, Chen X, Zhang Z, Jiang H, Yang L. Bistable insect-scale jumpers with tunable energy barriers for multimodal locomotion. Adv Sci. 2024;11(34):2404404. 10.1002/advs.202404404 PMC11425846 38973215

[39] Tang D, Zhang C, Pan C, Hu H, Sun H, Dai H, Fu J, Majidi C, Zhao P. Bistable soft jumper capable of fast response and high takeoff velocity. Sci Robot. 2024;9(93): Article eadm8484. 39167670 10.1126/scirobotics.adm8484

[40] Jiang Y, Li Y, Liu K, Zhang H, Tong X, Chen D, Wang L, Paik J. Ultra-tunable bistable structures for universal robotic applications. Cell Rep Phys Sci. 2023;4(5):101365.

[41] Hu N, Li B, Bai R, Xie K, Chen G. A torsion-bending antagonistic bistable actuator enables untethered crawling and swimming of miniature robots. Research. 2023;6:0116. 37287890 10.34133/research.0116 PMC10243200

[42] Deegan RD. Finessing the fracture energy barrier in ballistic seed dispersal. Proc Natl Acad Sci USA. 2012;109:5166–5169. 22431608 10.1073/pnas.1119737109 PMC3325694

[43] Pal A, Sitti M. Programmable mechanical devices through magnetically tunable bistable elements. Proc Natl Acad Sci USA. 2023;120(14): Article e2212489120. 37011212 10.1073/pnas.2212489120 PMC10104571

[44] Zhang Y, Velay-Lizancos M, Restrepo D, Mankame ND, Zavattieri PD. Architected material analogs for shape memory alloys. Matter. 2021;4:1990–2012.

[45] Niknam H, Akbarzadeh A, Therriault D, Bodkhe S. Tunable thermally bistable multi-material structure. Appl Mater Today. 2022;28:101529.

[46] Chen Z, Sun J, Zhao J. Tuning modules with elastic instabilities on-the-fly for reconfigurable shapes and motions. IEEE/ASME Trans Mechatron. 2024;29(4):3117–3127.

[47] Firouzeh A, Lee J, Yang H, Lee D, Cho K-J. Perching and grasping using a passive dynamic bioinspired gripper. IEEE Trans Robot. 2023;40:213–225.

[48] Sun J, Tighe B, Zhao J. Tuning the energy landscape of soft robots for fast and strong motion. In: 2020 IEEE International Conference on Robotics and Automation (ICRA). Paris (France): IEEE; 2020. p. 10082–10088.

[49] Yang D, Feng M, Sun J, Wei Y, Zou J, Zhu X, Gu G. Soft multifunctional bistable fabric mechanism for electronics-free autonomous robots. Sci Adv. 2025;11(5):eads8734. 39888988 10.1126/sciadv.ads8734 PMC11784860

[50] Yang Y, Fan L, Weng T, Zhao Y, Chen B, Li W. Bistable soft gripper with tension net applied to UAV. IEEE Robot Autom Lett. 2025;10(2):1920–1927.

[51] Wang X, Khara A, Chen C. A soft pneumatic bistable reinforced actuator bioinspired by Venus flytrap with enhanced grasping capability. Bioinspir Biomim. 2020;15(5): Article 056017. 32590362 10.1088/1748-3190/aba091

[52] Song N, Peng H, Guo X. Sym-ML: A symplectic machine learning framework for stable dynamic prediction of mechanical system. Mech Mach Theory. 2025;206: Article 105934.

[53] Zhang J, Li Y, Kan Z, Yuan Q, Rajabi H, Wu Z, Peng H, Wu J. A preprogrammable continuum robot inspired by elephant trunk for dexterous manipulation. Soft Robot. 2023;10(3):636–646. 36629865 10.1089/soro.2022.0048

[54] Zolfagharian A, Lakhi M, Ranjbar S, Sayah Irani M, Nafea M, Bodaghi M. 4D printing parameters optimisation for bi-stable soft robotic gripper design. J Braz Soc Mech Sci Eng. 2023;45: Article 224.

[55] Song N, Wang C, Peng H, Zhao J. A study of mechanism-data hybrid-driven method for multibody system via physics-informed neural network. Acta Mech Sin. 2025;41: Article 524159.

[56] Fang Q, Zhang J, He Y, Zheng N, Wang Y, Xiong R, Gong Z, Lu H. Drosophila larvae-inspired soft crawling robot with multimodal locomotion and versatile applications. Research. 2024;7:0357. 38716472 10.34133/research.0357 PMC11075670

[57] Mohammadi M, Kouzani AZ, Bodaghi M, Zolfagharian A. 3D-printed programmable bistable mechanisms for customized wearable devices in tremor attenuation. J Mech Behav Biomed Mater. 2025;168:107006. 40267690 10.1016/j.jmbbm.2025.107006

[58] Zolfagharian A, Demoly F, Lakhi M, Rolfe B, Bodaghi M. Bistable mechanisms 3D printing for mechanically programmable vibration control. Adv Eng Mater. 2025; Article 2402233.

[59] Zhang J, Yang H, Zhao Y, Yang J, Aydin YO, Li S, Rajabi H, Peng H, Wu J. Rapid, and stable trident robotic gripper: A bistable tensegrity structure implementation. IEEE/ASME Trans Mechatron. 2024.

[60] Geckeler C, Mintchev S. Bistable helical origami gripper for sensor placement on branches. Adv Intell Syst. 2022;4(10):2200087.

[61] Zhang C, Yang H, Garziera R, Xu Y, Jiang H. Reprogrammable gripper through pneumatic tunable bistable origami actuators. Int J Mech Sci. 2025;286: Article 109889.

