# Supplementary material for: Instant Energy Barrier Modulation in Bistable Robotic Grippers for Compliant Triggering and Powerful Grasping
Source: Research (Wash D C). 2025 Jun 19;8:0737. doi: 10.34133/research.0737 (PMC12178156; doi:10.34133/research.0737)
Supplement: Supplementary 1 — Figs. S1 to S14 Tables S1 and S2 [file research.0737.f1.zip › Supplementary Information.docx]

Supplementary Information for

**Instant energy barrier modulation in bistable robotic grippers for compliant triggering and powerful grasping**

**Authors:**

Jie Zhang, Hao Yang, Chenyu He, Hanfei Ma, Yuwen Zhao, Zongyu Zhang, Shengming Li, Wei Wang, Jinzhao Yang ^*^, Jianing Wu ^*^, Haijun Peng ^*^

*Corresponding author E-mail:

[yangjz3@mail.sysu.edu.cn](mailto:yangjz3@mail.sysu.edu.cn) (Jinzhao Yang)

[wujn27@mail.sysu.edu.cn](mailto:wujn27@mail.sysu.edu.cn) (Jianing Wu)

[hjpeng@dlut.edu.cn](mailto:hjpeng@dlut.edu.cn) (Haijun Peng)

**The file includes:**

**Supporting Notes**

Note S1: Effects of rotation angle on structural curvature

Note S2: Mechanical model of the curved beam

Note S3: Verification of mechanical model

Note S4. Duration of energy barrier modulation

Note S5. Cycle testing of the curved beam

**Supporting Figures**

Fig. S1: General design of our robotic gripper

Fig. S2: Physical dimension of the beam

Fig. S3: Theoretical critical curvature of the beam

Fig. S4: Effects of dropping height on response time.

Fig. S5: Measurement of Young’s modulus of the elastic beam

Fig. S6: Bending morphing of curved beam simulated by mechanical model and finite element method

Fig. S7: Experimental measurement of the forces *F*_t_ and *F*_f_ during grasping and detaching

Fig. S8: Effects of the object shape and material property on the triggering force

Fig. S9: Robotic gripper with low energy barriers grasping floating objects

Fig. S10: Comparison of maximum failure force *F*_f_ when adhering different types of sandpaper between the fingers

Fig. S11: Snapshots of the motor’s high-speed response

Fig. S12: Cycle testing of the curved beam

Fig. S13: Manipulating a diverse range of objects with varying shapes, sizes, and material properties

Fig. S14: High energy barrier enhancing carrying capacity of robotic gripper

**Supporting Tables**

Table S1: Comparison of energy barrier between theoretical model and FEA

Table S2: Performance comparison between our robotic gripper and other robotic grippers

**Supporting Videos**

Video S1: Stable state transition during grasping

Video S2: Triggering process of the robotic gripper

Video S3: Detachment process of the robotic gripper

Video S4: Robotic gripper with low energy barriers grasping floating objects

Video S5: Robotic gripper rapidly grasping marbles

Video S6: Automatic modulation of energy barrier in grasping process

Video S7: Effects of energy barrier modulation on carrying capacity

Video S8: UAV perching enabled by robotic gripper

**Note S1. Effects of rotation angle on structural curvature**

In the robotic gripper, the energy barriers can be tuned by altering the curvature of the beam (Fig. 2A). Here, we assume that the structural curvature of the beam is equal at any position during the deformation process. Since the curvature variation is achieved by the motor, we quantify the effects of rotation angle on the bending curvature, as showcased in Fig. 2B. In this figure, point *O* is used to describe the shaft of the motor, and point *ζ*_i_ (*i* = 1, 2) indicate the position of the slider before and after morphing. Additionally, points *P*_i_ and *Q*_i_ (*i* = 1, 2) are applied to denote the two ends of the curved beam, of which the length reaches *L*_d_.

When the beam is flat, the angle between the strut that connect the motor and the slider and the ground is denoted as *φ*, and the length of this strut *OS*_1_ is *l*. Then, the rotation of the motor causes this angle to increase by Δ*φ*. Consequently, the horizontal distance Δ*x*_s_ between the points *S*_1_ and *S*_2_ can be calculated as

|  |  | (S1) |
| --- | --- | --- |

Under such an actuation, the distance between the two ends (i.e., points *P*_2_ and *Q*_2_) of the curved beam with an angle of *θ* can be obtained.

|  | $\overline{P_{2}Q_{2}}=2L\sin\left(\theta/2\right)/\theta$ | (S2) |
| --- | --- | --- |

According to the geometrical relationship, we can obtain the following equation.

|  | $\overline{P_{2}Q_{2}}=L-2\Delta x_{s}$ | (S3) |
| --- | --- | --- |

Consequently, the bending angle *θ* of the curved beam can be calculated.

|  | $\theta=2\left(1/\left(1-\tau/L\right)^{2}-1\right)^{1/2}$   | (S4) |
| --- | --- | --- |

The curvature of the curved beam can be obtained.

|  | $\rho=\theta/L$ | (S5) |
| --- | --- | --- |

**Note S2. Mechanical model of the curved beam**

The elastic beam is discretized into a series of Euler-Bernoulli beam elements, as shown in Fig. S6A. For the *i*^th^ node, the generalized coordinate ***q***_i_ is defined as

|  | $q_{i}=\left[\begin{matrix} r_{i}^{T} & a_{i}^{T} & \varepsilon_{i}^{T} \end{matrix}\right]^{T}$ | (S6) |
| --- | --- | --- |

where, ***r***_i_ represents the coordinate of the *i*^th^ node in the global coordinate system, ***a***_i_ denotes the unit quaternion, and ***ε***_i_ is the normal strain at the *i*^th^ node.

The coordinate of the centerline of the *j*^th^ element can be expressed by the Hermite interpolation, as

|  | $\bar{r}_{i}\left(\xi\right)=N_{1}r_{j}+N_{2}l_{e}\left(1+\varepsilon_{j}\right)t_{j}+N_{3}r_{j+1}+N_{4}l_{e}\left(1+\varepsilon_{j+1}\right)t_{j+1}$ | (S7) |
| --- | --- | --- |

where,

|  | $\left\{\begin{matrix} N_{1}\left(\xi\right)=1-3\xi^{2}+2\xi^{3} \\ \begin{matrix} N_{2}\left(\xi\right)=\xi\left(1-\xi\right)^{2} \\ N_{3}\left(\xi\right)=\xi^{2}\left(3-2\xi\right) \\ N_{4}\left(\xi\right)=\xi^{2}\left(\xi-1\right) \end{matrix} \end{matrix}\right.$ | (S8) |
| --- | --- | --- |

are Hermite shape functions. ζ ∈ [0,1] is the normalized arc-length parameter. *l*_e_ is the element length. ***t***_i_ denotes the unit tangent vector of the centerline at *i*^th^ node and can be given by

|  | $t_{i}=\left[\begin{matrix} \left(a_{i,0}^{2}+a_{i,1}^{2}\right)-\left(a_{i,2}^{2}+a_{i,3}^{2}\right) \\ 2\left(a_{i,1}a_{i,2}+a_{i,0}a_{i,3}\right) \\ 2\left(a_{i,1}a_{i,3}-a_{i,0}a_{i,2}\right) \end{matrix}\right]$ | (S9) |
| --- | --- | --- |

After the elastic beam is deformed by the external force, the normal strain at ζ on the *j*^th^ element can be calculated by

|  | $\bar{\varepsilon}_{i}\left(\xi\right)=\bar{t}_{i}^{T}\bar{r}_{i}^{'}/l_{e}-1$ | (S10) |
| --- | --- | --- |

where, the notation represents the derivative with respective to the normalized arc-length parameter ζ and can be expressed as

|  | $\bar{t}_{i}\left(\xi\right)=\bar{r}_{i}^{'}/\left\Vert\bar{r}_{i}^{'}\right\Vert$ | (S11) |
| --- | --- | --- |

Additionally, the curvature at ζ on the *j*^th^ element can be expressed as

|  |  | (S12) |
| --- | --- | --- |

Since the constitutive relation of the elastic beam is linear elasticity, the strain energy of the *j*^th^ element can be calculated by

|  | $U_{i}=\frac{l_{e}}{2}\int_{0}^{1} \left(EA\bar{\varepsilon}_{i}^{2}+GJ_{1}\bar{\chi}_{i,1}^{2}+EI_{2}\bar{\chi}_{i,2}^{2}+EI_{3}\bar{\chi}_{i,3}^{2}\right)d\xi$ | (S13) |
| --- | --- | --- |

where, *E* and *G* are Young’s modulus and the shear modulus, respectively. *A* is the cross-sectional area. *J* = diag(*J*_1_, *I*_2_, *I*_3_) is the moment of inertia of the cross-section. Hence, the total strain energy of the elastic beam can be expressed as

|  | $U=\sum_{i} U_{i}$ | (S14) |
| --- | --- | --- |

After deriving the elastic strain energy of the system, the mechanical model of the elastic beam can be formulated as a set of nonlinear algebraic equations [S1]:

|  | $\left\{\begin{matrix} \partial E/\partial q+\left(\partial C/\partial q\right)^{T}\lambda=f \\ C=0 \end{matrix}\right.$ | (S15) |
| --- | --- | --- |

where, *E* denotes the elastic strain energy, ***q*** is the generalized coordinate of the elastic beam, ***λ*** denotes the Lagrange multiplier, ***f*** is the external force, and ***C*** represents the constraint equation consisting of both the unit quaternion constraints and the boundary conditions.

The boundary condition rotating the ends of the elastic beam around the rotating shafts (i.e., *z*-axis) can be expressed as

|  | $\left\{\begin{matrix} r_{b}-r_{b,0}=0 \\ a_{b}^{2}-1=0 \\ a_{b,1}=0 \\ a_{b,3}=0 \\ 2\arcsin\left(a_{b,2}\right)-\left(2\arcsin\left(a_{b,2,0}\right)+\gamma\right)=0 \end{matrix}\right.$ | (S16) |
| --- | --- | --- |

where, subscript *b* represents number 0 and/or *n*, *r*_b,0_ denotes the coordinate of the node *b* at the initial time, *a*_b,2,0_ denotes the initial quaternion *a*_b,2_, and *γ*_b_ is the applied rotation angle. The mechanical model of the elastic beam, namely Eq. (S15), can be solved by the Newton-Raphson algorithm.

Additionally, the boundary condition for moving the two ends of the elastic curved beam towards the middle can be expressed as

|  | $\left\{\begin{matrix} r_{b}-d_{s}=0 \\ a_{b}-a_{b,0}=0 \end{matrix}\right.$ | (S17) |
| --- | --- | --- |

where, ***d***_b_ denotes the distance that the ends of the elastic beam needed to be moved, and ***a***_b,0_ represents the initial quaternion ***a***_b_ at the ends of the beam.

**Note S3. Verification of mechanical model**

To validate the accuracy of the mechanical model, finite element simulations are performed by using commercially available software Ansys Workbench to quantify the bending deformation of the curved beam. In the simulations, the material (i.e., PET) of the beam is assumed to be elastic, while other components are treated as rigid. Young’s modulus and Poisson's ratio of PET are set as 3364 MPa and 0.39, respectively. Here, the Young’s modulus is measured through stress-strain curves, as showcased in Fig. S5. These parameters are also employed to mechanical models. Subsequently, the beam is meshed using 8-node first-order hexahedral elements with an average size of 0.50 mm. The LS-DYNA module is used for implicit solving to simulate state transitions. The simulation is divided into the following two steps. Step 1: we fix one of the supports and move the other inward to induce bending in initially the flat beam. In this stage, the displacement *L*_D_ of the beam reaches 13.31 mm, which is approximately consistent with the results obtained from finite element simulation (13.95 mm), as shown in Fig. S6B. Step 2: Both supports are fixed and a downward displacement is applied to the middle of the curved beam. For this snap-through, the energy barriers are 9.39 and 10.11 mJ, as shown in Fig. S6C and S6D. The subtle difference between these above results may be due to the failure to consider the constraint effect of the clamps in the mechanical model. The consistency of the results illustrates that our mechanical model can be applied to predict curvature deformation and energy barriers. Subsequently, the elastic energy of the system can be enhanced by shortening the distance *L*_d_ between the two ends of the curved beam (Fig. S6E).

**Note S4.** **Duration of energy barrier modulation**

Here, the sampling rate of the sensors is 80 ms. To improve its anti-interference ability, it is only considered that the bistable structure has undergone a state transition when the results within three sampling periods are zero. Subsequently, the transition of this signal is applied as input to modulate the distance *L*_d_ between the supports, increasing in the energy barrier of the bistable robotic gripper. According to our experiments, the regulation duration of the motor is 60 ms (Fig. S11). Therefore, the regulation of the energy barrier requires a delay of ~300 ms.

**Note S5. Cycle testing of the curved beam**

Three reflective markers are attached to the curved beam. Then, an optical motion capture system (Prime 13, OptiTrack, USA) is applied to capture the displacement of marked points in the deformation process of the curved beam. The sampling frequency of the system is 240 fps.

| 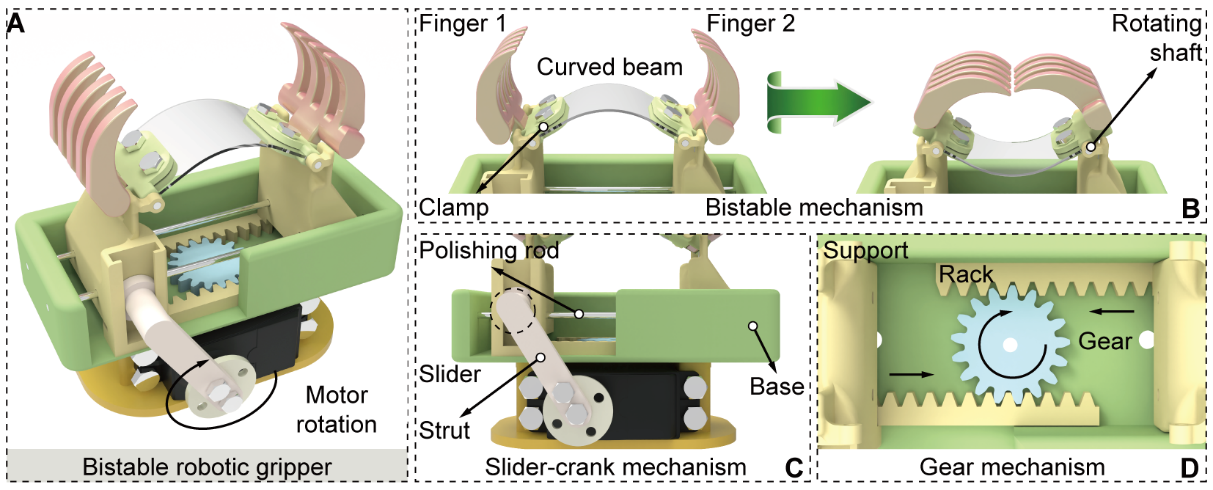 |
| --- |
| **Fig. S1. General design of our robotic gripper.** (A) Assembly of the bistable robotic gripper, in which three diverse mechanisms are incorporated, involving (B) bistable mechanism, (C) slider-crank mechanism, and (D) gear mechanism. |

| 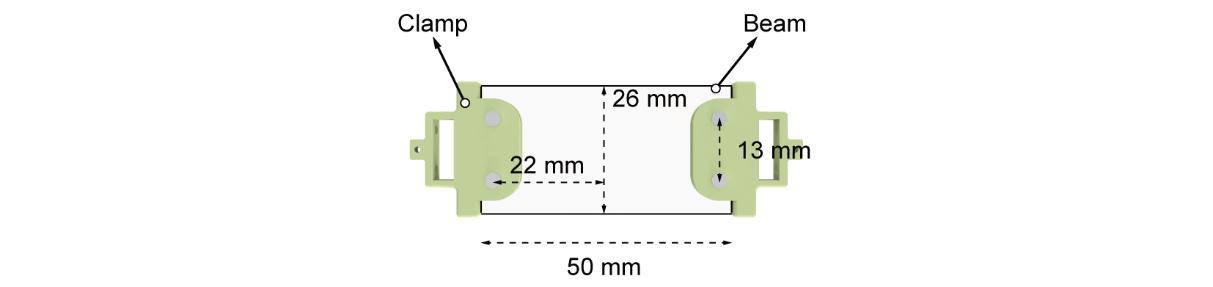 |
| --- |
| **Fig. S2.** **Physical dimension of the beam.** The physical dimensions of this beam are 50*26*0.20 mm. |

| 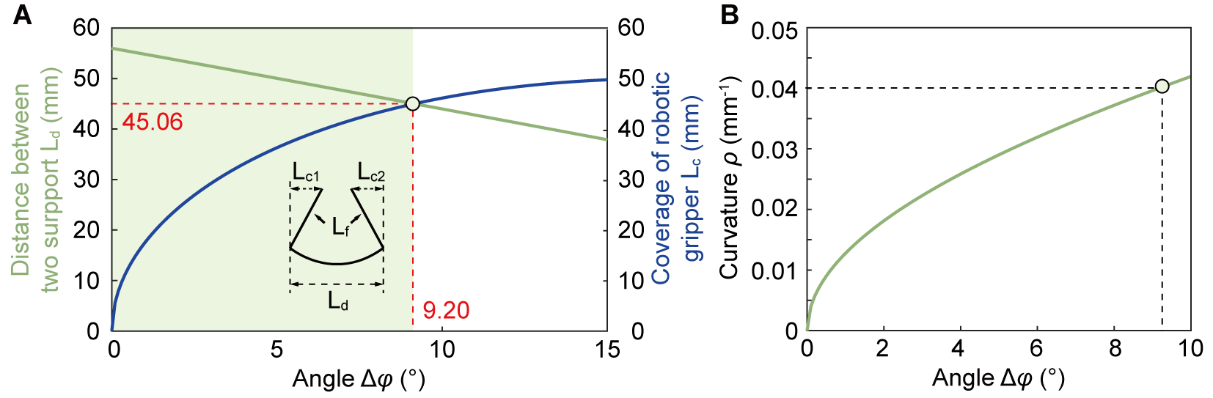 |
| --- |
| **Fig. S3.** **Theoretical critical curvature of the beam.** (A) Effects of rotation angle Δ*φ* on both distance *L*_d_ and coverage *L*_c_. In this robotic gripper, *L*_c_ equals the sum of *L*_c1_ and *L*_c2_. (B) Effects of rotation angle Δ*φ* on the curvature. |

| 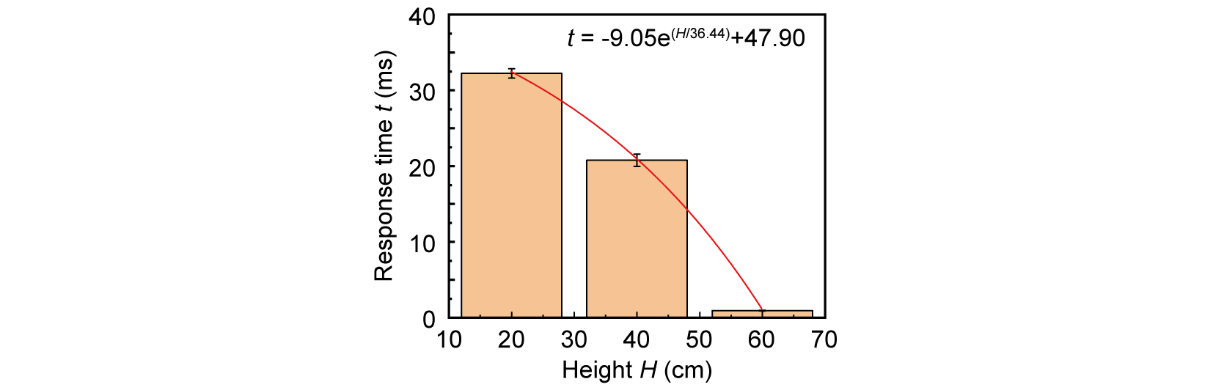 |
| --- |
| **Fig. S4. Effects of dropping height *H* on response time *t*.** |

| 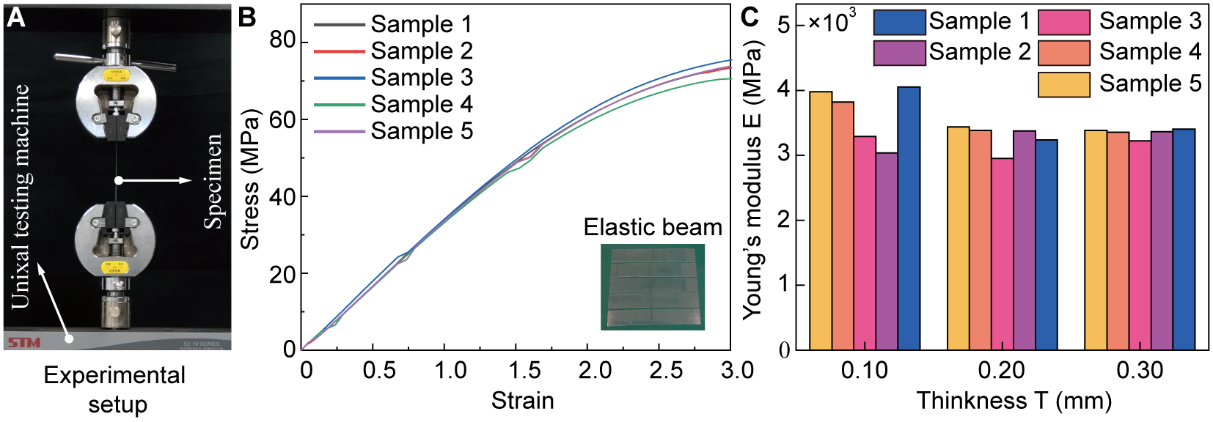 |
| --- |
| **Fig. S5.** **Measurement of Young’s modulus of the elastic beam.** (A) Experimental setup. (B) Stress-strain curve of the elastic beam. (C) Young's modulus of the elastic beams with diverse thicknesses. |

| 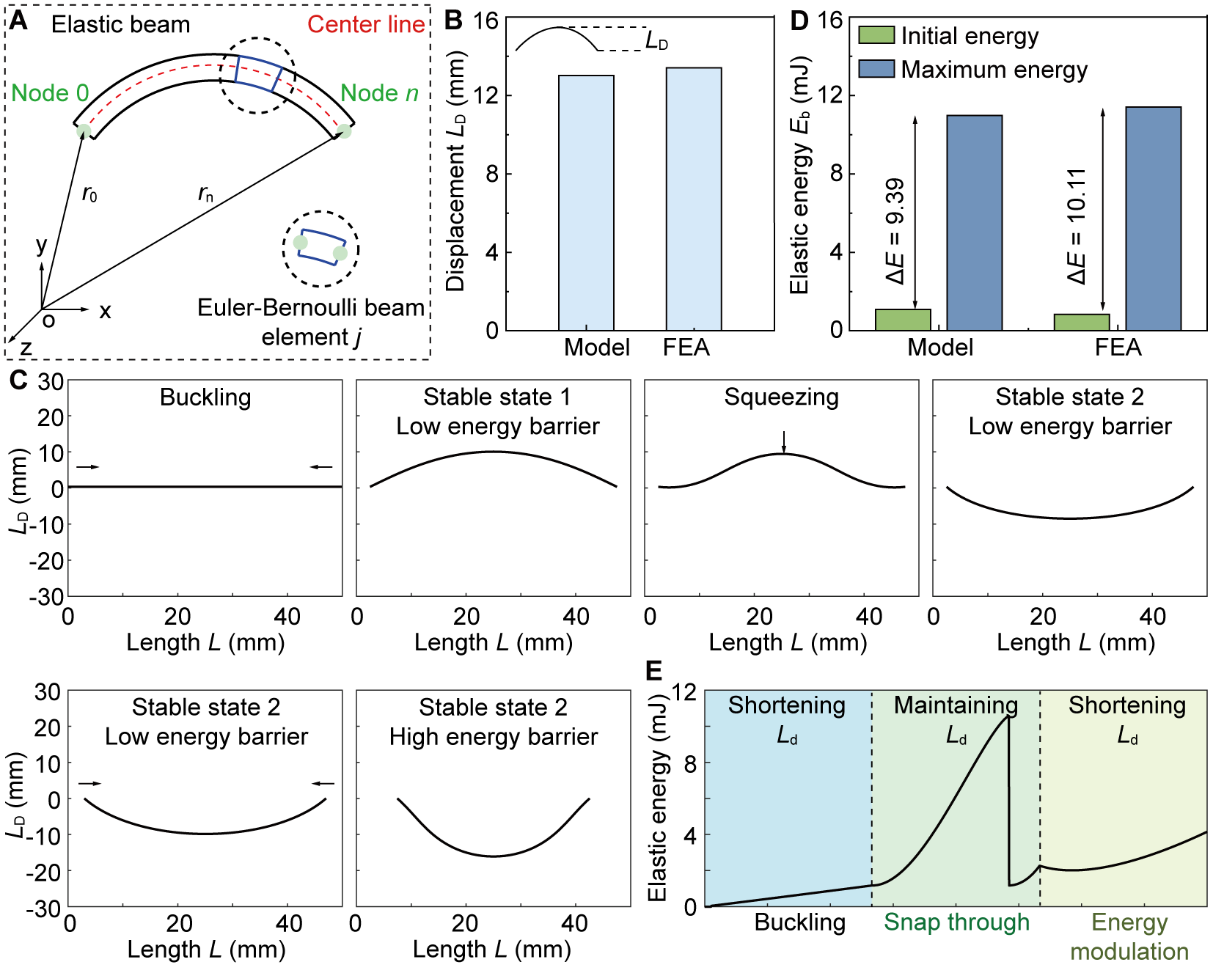 |
| --- |
| **Fig. S6. Bending morphing of curved beam simulated by mechanical model and finite element method.** (A) Modeling diagram of the beam. (B) Initial displacement *L*_D_ of the curved beam. (C) Simulation of morphing process of the curved beam. (D) Comparison of both elastic energy and energy barrier between mechanical model and finite element analysis. (E) Diagram of elastic energy variation in our robotic gripper. |

| 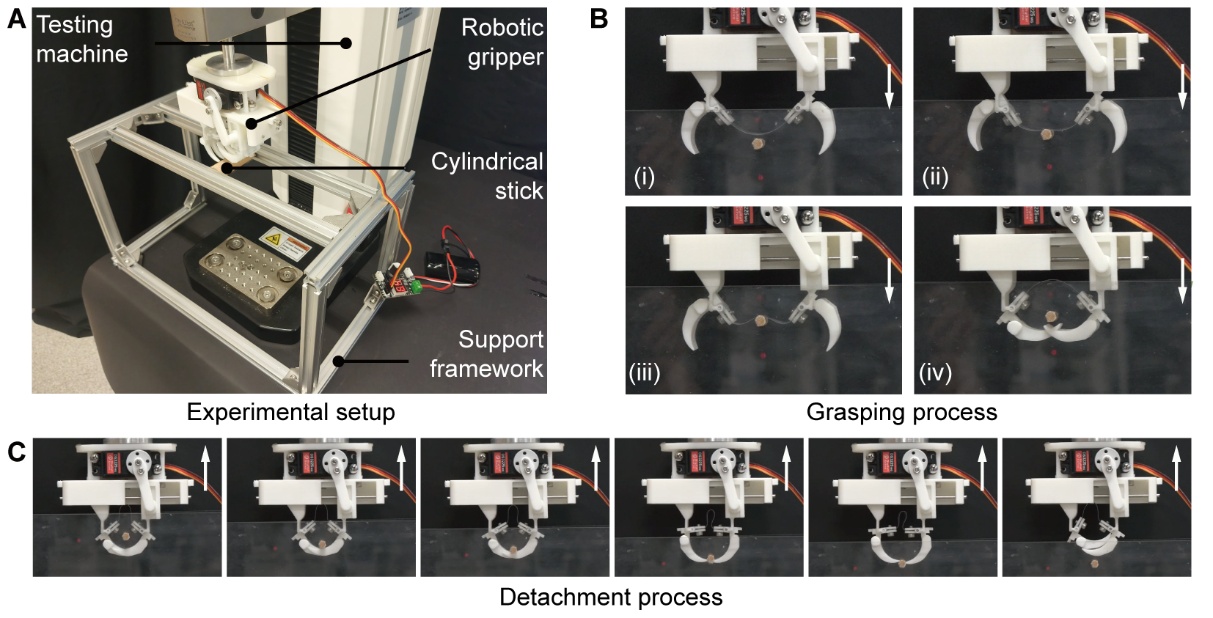 |
| --- |
| **Fig. S7.** **Experimental measurement of the forces *F*_t_ and *F*_f_ during grasping and detaching.** (A) Experimental setup. (B) Triggering snapshots of our robotic gripper. (C) Detachment process of the robotic gripper. |

| 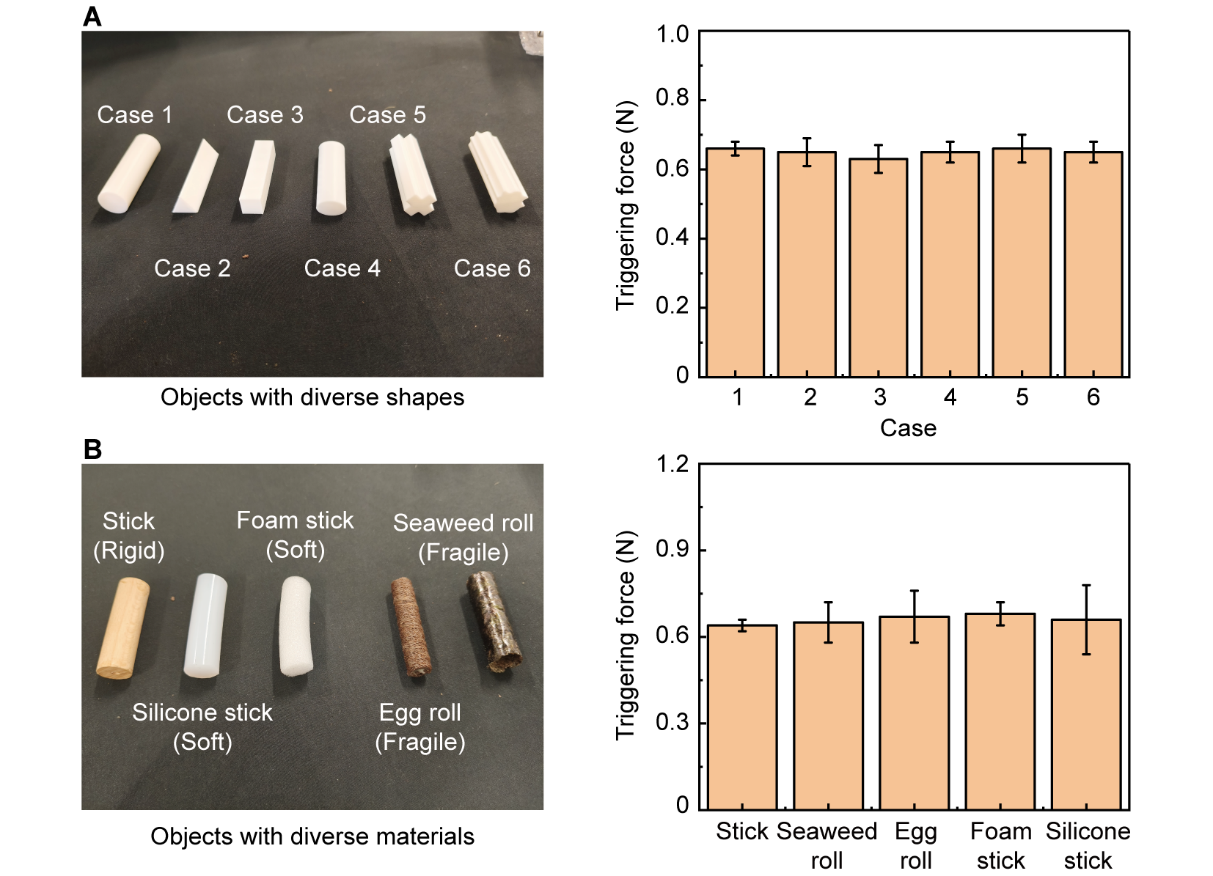 |
| --- |
| **Fig. S8. Effects of the object shape and material property on the triggering force.** (A) Objects with diverse shapes, such as triangular prism, quadrangular, and elliptic cylinder. (B) Objects with diverse material properties, such as rigid, soft, and fragile characteristics. |

| 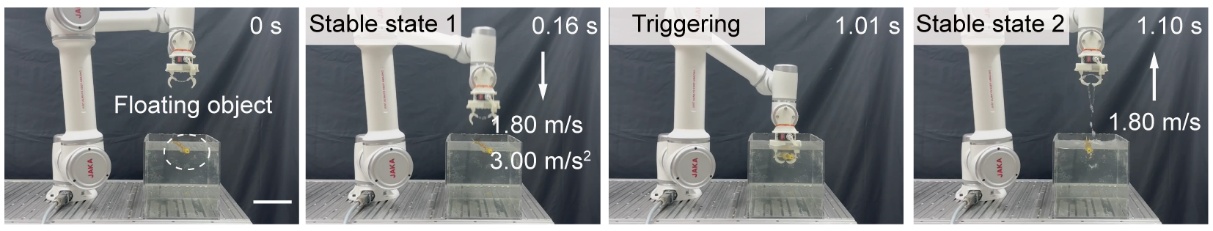 |
| --- |
| **Fig. S9. Robotic gripper with low energy barriers grasping floating objects.** |

| 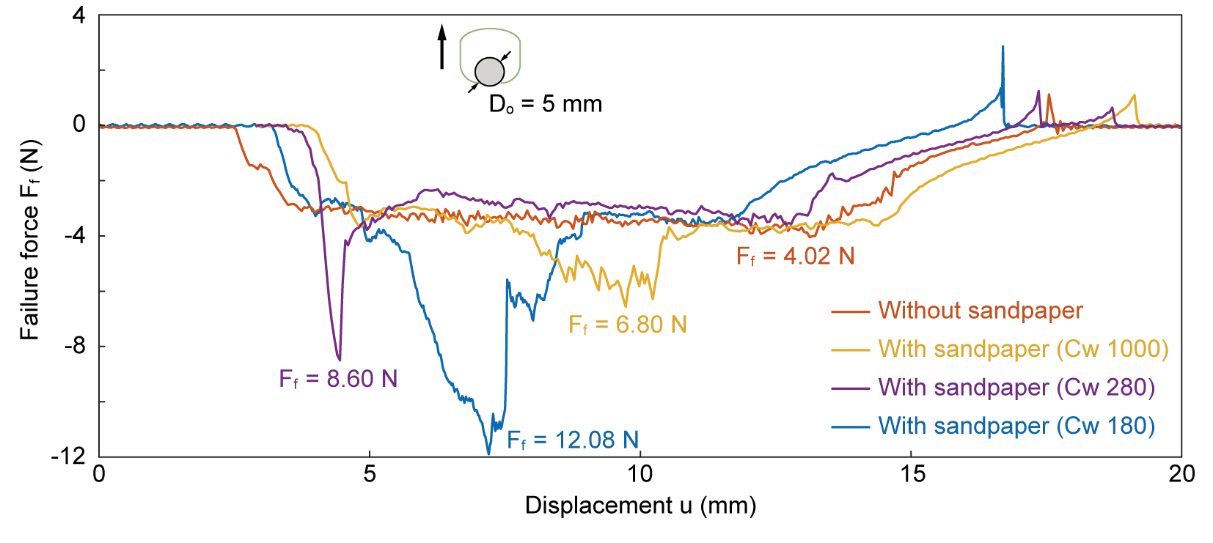 |
| --- |
| **Fig. S10.** **Comparison of maximum failure force *F*_f_ when adhering different types of sandpaper between the fingers.** The diameter of the cylindrical stick is 5 mm. |

| 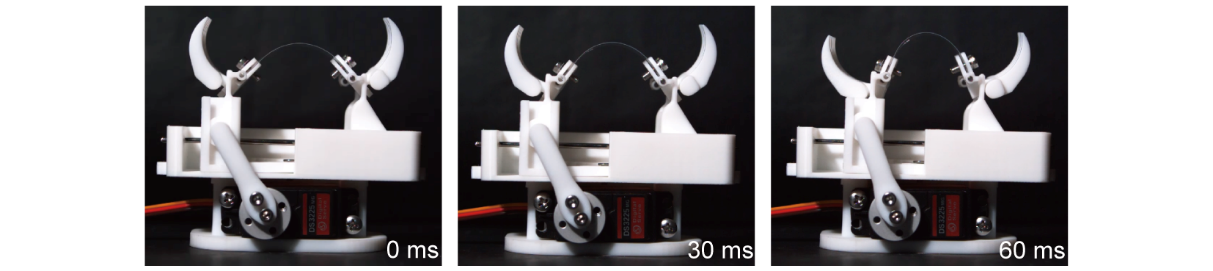 |
| --- |
| **Fig. S11. Snapshots of the motor’s high-speed response.** |

| 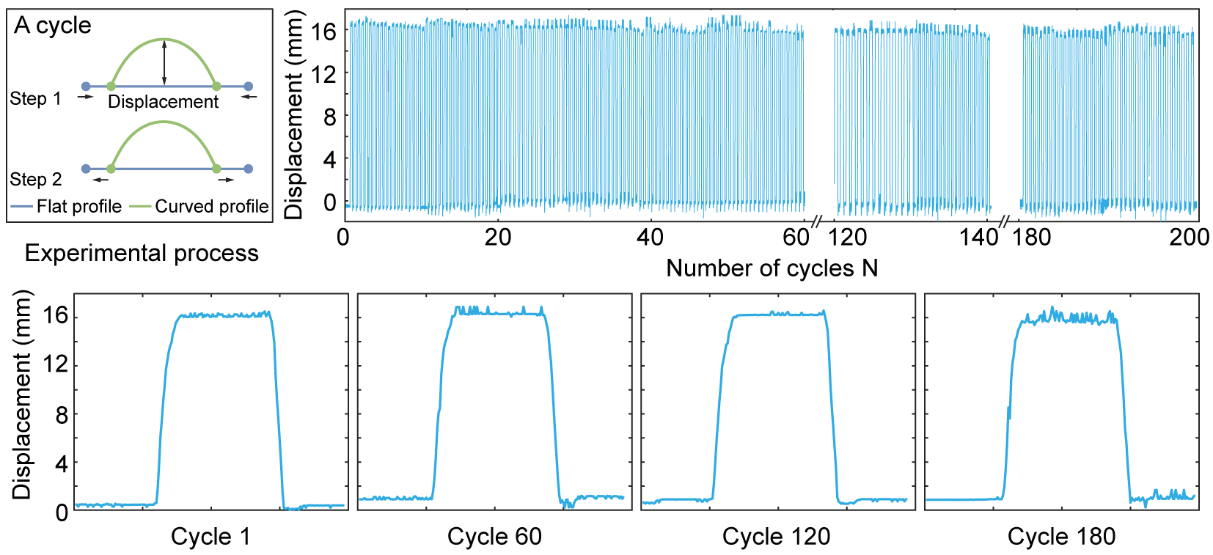 |
| --- |
| **Fig. S12. Cycle testing of the curved beam.** |

| 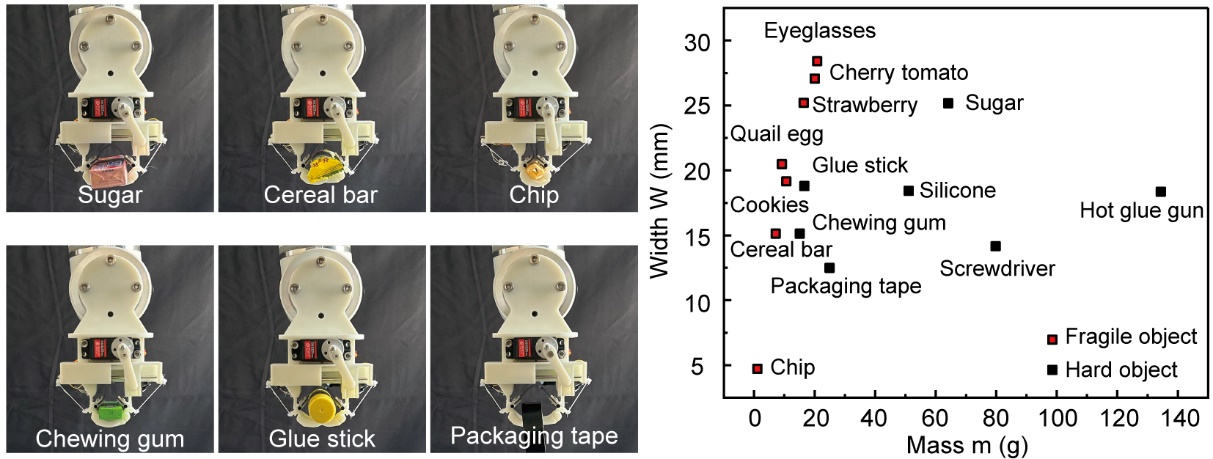 |
| --- |
| **Fig. S13.** **Manipulating a diverse range of objects with varying shapes, sizes, and material properties.** Both mass and physical dimension of the objects are showcased in the figure. |

| 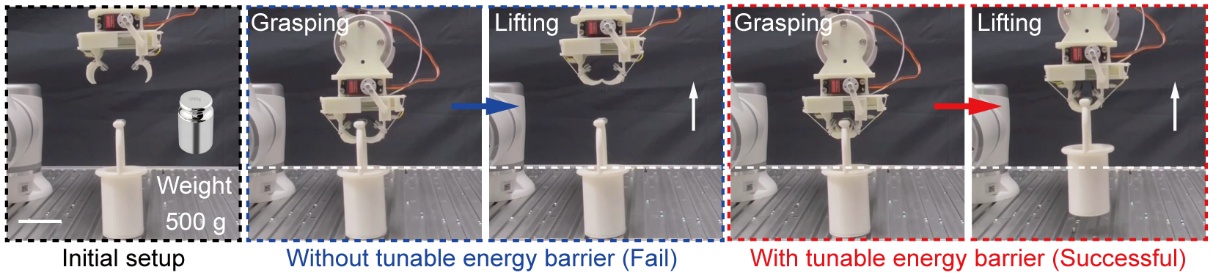 |
| --- |
| **Fig. S14. High energy barrier enhancing carrying capacity of robotic gripper.** |

**Table S1. Comparison of energy barrier between theoretical model and FEA**

| Method | Model | FEA | Model | | FEA | Model | | FEA |
| --- | --- | --- | --- | --- | --- | --- | --- | --- |
| Distance  *L*_d_ (mm) | 35 | 35 | 40 | 40 | | 45 | 40 | |
| Initial energy  *E*_1_ (mJ) | 3.72 | 3.48 | 2.41 | | 2.13 | 1.17 | | 0.80 |
| Maximum energy  *E*_2_ (mJ) | 33.53 | 35.42 | 21.71 | | 22.74 | 10.56 | | 10.91 |
| Energy barrier  *E* (mJ) | 29.81 | 31.94 | 19.30 | | 20.61 | 9.39 | | 10.11 |
| **Relative error** | **6.67%** | | **6.35%** | | | **7.12%** | | |

**Table S2.** **Performance comparison between our robotic gripper and other robotic grippers**

| Reference | Active actuator | Energy tunability | Triggering force (N) | Failure force (N) | Ratio | Duration (ms) | Mass (g) |
| --- | --- | --- | --- | --- | --- | --- | --- |
| [47] | No | No | 1.00 | 0.50 | 0.50 | 186 | 56.33 |
| [48] | No | No | 1.50 | 0.34 | 0.23 | 800 | 4.20 |
| [49] | No | No | 1.57 | 2.80 | 1.78 | 1730 | 5.00 |
| [23] | Yes | Yes | 1.50 | 10.00 | 6.70 | 80 | >1000 |
| [50] | Yes | Yes | 40.00 | 23.75 | 0.59 | 110 | 536 |
| [51] | Yes | Yes | 4.00 | 6.00 | 1.50 | 50 | - |
| [52] | Yes | Yes | 5.00 | 3.44 | 0.69 | 130 | - |
| [25] | Yes | Yes | 8.50 | 24.88 | 2.93 | 300 | 35 |
| **This work**  **(without friction)** | **Yes** | **Yes** | **0.66** | **5.56** | **8.42** | **25** | **150** |
| **This work**  **(with friction)** | **Yes** | **Yes** | **0.66** | **12.08** | **18.18** | **25** | **150** |

**Reference**

[S1] Zhao Z H, Ren G X. A quaternion-based formulation of Euler-Bernoulli beam without singularity [J]. Nonlinear Dynamics, 2012, 67(3): 1825-1835.
